# Epigenetic Vulnerability of Insulator CTCF Motifs at Parkinson’s Disease-Associated Genes in Response to Neurotoxicant Rotenone

**DOI:** 10.3389/fgene.2020.00627

**Published:** 2020-07-07

**Authors:** Dana M. Freeman, Zhibin Wang

**Affiliations:** Laboratory of Environmental Epigenomes, Department of Environmental Health and Engineering, Bloomberg School of Public Health, Johns Hopkins University, Baltimore, MD, United States

**Keywords:** CTCF, Parkinson’s disease, single nucleotide polymorphisms, rotenone, DNA methylation, histone modifications

## Abstract

CCCTC-binding factor (CTCF) is a regulatory protein that binds DNA to control spatial organization and transcription. The sequence-specific binding of CTCF is variable and is impacted by nearby epigenetic patterns. It has been demonstrated that non-coding genetic variants cluster with CTCF sites in topological associating domains and thus can affect CTCF activity on gene expression. Therefore, environmental factors that alter epigenetic patterns at CTCF binding sites may dictate the interaction of non-coding genetic variants with regulatory proteins. To test this mechanism, we treated human cell line HEK293 with rotenone for 24 h and characterized its effect on global epigenetic patterns specifically at regulatory regions of Parkinson’s disease (PD) risk loci. We used RNA sequencing to examine changes in global transcription and identified over 2000 differentially expressed genes (DEGs, >1.5-fold change, FDR < 0.05). Among these DEGs, 13 were identified as PD-associated genes according to Genome-wide association studies meta-data. We focused on eight genes that have non-coding risk variants and a prominent CTCF binding site. We analyzed methylation of a total of 165 CGs surrounding CTCF binding sites and detected differential methylation (|>1%|, *q* < 0.05) in 45 CGs at 7 PD-associated genes. Of these 45 CGs, 47% were hypomethylated and 53% were hypermethylated. Interestingly, 5 out of the 7 genes had correlated gene upregulation with CG hypermethylation at CTCF and gene downregulation with CG hypomethylation at CTCF. We also investigated active H3K27ac surrounding the same CTCF binding sites within these seven genes. We observed a significant increase in H3K27ac in four genes (FDR < 0.05). Three genes (PARK2, GPRIN3, FER) showed increased CTCF binding in response to rotenone. Our data indicate that rotenone alters regulatory regions of PD-associated genes through changes in epigenetic patterns, and these changes impact high-order chromatin organization to increase the influence of non-coding variants on genome integrity and cellular survival.

## Introduction

Parkinson’s disease (PD) is the second most common neurodegenerative disorder in the United States (de Lau and Breteler, 2006). More than 800 genetic association studies have been conducted to interpret genetic contribution to PD etiology (Lill et al., 2012; Coetzee et al., 2016). Genome-wide association studies (GWAS) evaluate the association of common genetic variants to a phenotype or disease outcome. Since 2005, thousands of variants have been identified to have a significant association with a disease and more than 1600 single-nucleotide polymorphisms (SNPs) have been identified as genetic risk variants for PD (Lill et al., 2012). However, unlike rare monogenetic associations, the functional consequences of most of these variants have yet to be determined. Over 90% of all indexed SNPs including those associated with PD occur in non-coding regions of the genome (Maurano et al., 2012; Verstraeten et al., 2015). This discovery led to the hypothesis that SNPs in the human genome interact with regulatory elements to control gene expression (Wang et al., 2019). This is supported by expression of quantitative trait loci (eQTLs) defined as genetic regions that are enriched at positive GWAS sites and explain variability in the expressivity of a gene (Nica et al., 2010). Despite these advances, it remains a challenge to determine which genetic variants in a broad region of variants is the driver of gene expression changes particularly when regulatory element interactions are long-range (Do et al., 2016). SNPs cluster within enhancers and can modify PD risk. These observations of PD-associated SNPs have been described in multiple cell types (Coetzee et al., 2016). With this new evidence, studies are now focusing on interactions of regulatory elements to understand how genetic associations trigger disease biology within the brain.

CCCTC-binding factor can play a long-range cis-regulatory role that insulates genes from their surrounding signaling environment by directing chromatin looping (Phillips and Corces, 2009). Functional CTCF binding sites are required for the formation of distinct structural domains within a three-dimensional chromosomal organization (Ong and Corces, 2014; Tang et al., 2015). CTCF binding is dependent upon DNA sequence (CCGCGNGGNGGCAG) and allelic hypomethylation (Wang et al., 2012). Thus, genetic variants and epigenetic patterns within binding sites can contribute to dysfunctional CTCF allele-specific binding (Tang et al., 2015; Wang et al., 2019).

Approximately 85% of PD cases cannot be explained by genetic predisposition alone (Franco et al., 2010; Verstraeten et al., 2015; Labbé et al., 2016). Therefore, it is likely that most cases are caused by the interplay of common SNPs with environmental factors. Environmental factors can modulate the association of a genetic variant with a disease (Lee et al., 2011). For instance, exposures that impact allele-specific methylated regions in the genome can influence CTCF binding and thus influence non-coding variants’ effect on genetic expression (Wang et al., 2019). GWAS association signals are complex in that they can cover a broad region of DNA with several polymorphisms, so we focused on environmentally induced epigenetic changes in CTCF binding regions nearby risk-associated genes to explore mechanisms of gene–environment interactions in PD.

In our pesticide-induced cellular model, we used rotenone, a naturally occurring insecticide and potent inhibitor of complex I in the mitochondrial electron transport chain. The primary use of rotenone today is as a piscicide to terminate invasive or noxious species of fish. Permissible application concentrations up to 250 ppb can be applied to public and recreational waters (United States Environmental Protection Agency [USEPA], 2007). Rotenone is a widely accepted PD toxicant and can robustly replicate pathology via depletion of ATP, generation of reactive oxygen species, damage of nigrostriatal tissues, and death of dopamine producing cells in the midbrain (Dawson et al., 2002; Cicchetti et al., 2009). It has also been shown to cause these types of cellular pathology in HEK293 (Orth et al., 2003; Teixeira et al., 2018). While cell line HEK293 is a human immortalized cell line derived originally from primary embryonic kidney cells, it has been found to have a genetic signature similar to neurons (Stepanenko and Dmitrenko, 2015). We chose this cell line given their well-characterized genome and ENCODE regulatory elements (ENCODE Project Consortium, 2012; Lin et al., 2014).

DNA methylation and histone acetylation are epigenetic modifications implicated in rotenone-induced neurotoxicity (Huang et al., 2019). DNA hypomethylation has been reported in response to pesticide exposure (Hou et al., 2012), and we discovered that rotenone reduces DNA methylation at DNMT1-dependent regions in the human genome (Freeman et al., 2020). Histone acetylation patterns have been more extensively studied in rotenone-induced PD due to its high correlation with gene expression and enhancer activation (Wang et al., 2008). Most studies agree that rotenone-induced neurodegeneration is associated with pathological hyperacetylation as a result of impaired homeostatic activity of HATs and HDACs (Feng et al., 2015; Park et al., 2016; Harrison et al., 2018; Wang et al., 2018; Huang et al., 2019).

In this study, we examined rotenone-induced changes in DNA methylation and histone acetylation patterns at CTCF binding sites adjacent to PD-associated genes. Eight selected genes had identified disease-risk SNPs in a non-coding region and were indexed by a meta-data analysis of over seven million human polymorphisms (Lill et al., 2012). We hypothesize that rotenone exposure modifies epigenetic patterns at CTCF binding motifs and affects its allele-specific transcription factor binding. We postulate that this mechanism could mediate the interchange between genetic variants and regulatory elements controlling transcription and genomic stability.

## Materials and Methods

### Cell Culture and Treatment of Human Cell Line HEK293

All media reagents and chemicals in cell culture were purchased from Sigma (St. Louis, MO, United States). Human cell line HEK293 was grown in Dulbecco’s Modified Eagle Medium with high glucose, L-glutamine, and sodium pyruvate. Media were supplemented with 10% (v/v) heat-inactivated fetal bovine serum and 1% (v/v) penicillin–streptomycin. HEK293 cells were confirmed by ATCC. Cells were treated at approximately 70% confluency with 200 nM rotenone or DMSO vehicle control (<0.001%) for 24 h. Cell viability was measured with trypan blue (0.4%) staining, and cells were counted manually with a hemocytometer. Cells used for experiments were at least 85% viable relative to the vehicle control.

### Qualitative Analysis of Total 5mC Levels

Genomic DNA was extracted from two replicates of DMSO or rotenone-treated HEK293 using 1:1:1 phenol: chloroform: isoamyl alcohol (Sigma, St. Louis, MO, United States). Global DNA methylation was first measured by dot blot analysis. Bisulfite-treated DNA (30–60 ng/μL) was denatured at 95°C for 5 min and then cooled at 4°C for 5 min in a conventional thermocycler (MyCycler; Bio-Rad; Hercules, CA, United States). DNA was spotted onto 0.45-micron nitrocellulose paper as 1- or 2-μL drops and dried for 30 min at room temperature. The membrane was UV cross-linked at 3000 Hz and incubated in anti-5methylcytosine (5mC) primary antibody overnight at 4°C (Epigentek 33D3; Farmingdale, NY, United States). The membrane was washed with TBST and incubated with a secondary antibody conjugated to HRP for 1 h at room temperature (Santa-Cruz Biotechnology anti-mouse IgG sc-2005; Dallas, TX, United States). The membrane was washed again with TBST after secondary incubation and visualized with chemiluminescence (ProSignal Femto; Prometheus; Raleigh, NC, United States). We used a serial dilution of 100% 5mC standard (Zymo Research D5012; Irvine, CA, United States) to create a standard ladder (Supplementary Figure 1).

### HEK293 Western Blot for Global H3K27ac

HEK293 cells were collected after a 24-h treatment, and histones were extracted using the Abcam Histone Extraction kit according to kit instructions (Cambridge, United Kingdom). Histone protein concentration was measured by Qubit Protein Assay from Thermo Fisher Scientific (Waltham, MA, United States). Protein (5 μg) was loaded onto 4–15% Bio-Rad Page Gels and transferred to 0.45 μm nitrocellulose (Bio-Rad, Hercules, CA, United States). The blots were incubated with H3K27ac primary antibody (1:1000; Abcam ab4729) overnight at 4°C and anti-rabbit IgG conjugated secondary antibody (1:5000, Santa Cruz Biotechnology Sc-2357) for 1 h at room temperature. The histone protein was normalized to total histone 3 (H3; Abcam ab1791) and quantified with ImageJ software. We tested significance by comparing the ratio of H3K27ac/H3 with a two-tailed Student’s paired *t*-test (*p* < 0.05).

### RNA Extraction and RNA Sequencing Library Construction

Total RNA was extracted from two replicates of DMSO or rotenone-treated HEK293 using the TRIzol method (Invitrogen, Carlsbad, CA, United States). A total of 2 μg per sample was used for library construction using the TruSeq Sample Preparation kit from Illumina (San Diego, CA, United States). Poly-A-containing mRNA molecules were isolated from total RNA using oligo-dT attached magnetic beads. Isolated mRNA was then fragmented and synthesized into double-stranded cDNA according to kit instructions. Ligation of unique Illumina adapter indices was completed for each sample before bead purification. Libraries were loaded onto a 2% agarose gel, and library products between 200–800 bp were purified using the mini-Elute gel extraction kit from Qiagen (Hilden, Germany). Approximately 150 ng was sent for sequencing on a HiSeq 2000 platform with 100-bp paired-end reads.

### RNA Sequencing Data Analysis

Adapter sequences were removed from the raw sequencing data, and individual libraries were converted to the fastq format. Sequencing reads were aligned to the human genome (hg19) with TopHat2 (v2.0.9) (Kim et al., 2013). For mRNA analyses, the RefSeq database (Build 37.3) was chosen as the annotation references. Read counts of annotated genes were obtained by the Python software HTSeq count (Anders et al., 2015). DEGs were defined as those with a 1.5-fold change in expression using FDR < 0.05 from the edgeR package (Robinson et al., 2010). Gene Ontology annotation was done with Gorilla online platform and visualized with Revigo and Cytoscape (Eden et al., 2009; Supek et al., 2011; Otasek et al., 2019).

### RNA Sequencing Validation With Quantitative Reverse Transcription-PCR

Total RNA was extracted from an additional replicate of HEK293 treated with DMSO or rotenone using the same procedure as stated above. A total of 500 ng RNA was converted to cDNA with the PrimeScript RT reagent kit with gDNA eraser from Takara (Kusatsu, Japan). We selected 10 genes for quantitative PCR (qPCR) analysis using primers listed in Supplementary Table 1. All qPCR reactions were performed on a 7500 Real-Time PCR system from Applied Biosystems (Foster City, CA, United States) using the iTaq Universal SYBR Green Supermix from Bio-Rad (Hercules, CA, United States). The change in expression was normalized to the GAPDH housekeeping gene and expressed as fold change (2^–ΔΔCT^).

### Identification and Selection of PD-Associated Genes

We identified PD-associated genes using the National Health Genomic Research Institute GWAS Catalog (Buniello et al., 2018). We searched for all associations both reported and mapped using the trait “Parkinson’s disease” (EFO_0002508) which included 39 publications investigating genomic signatures of both familial and environmentally driven PD as well as Lewy body pathology and Parkinsonism in frontotemporal lobe dementia (Supplementary File 2). We calculated the frequency for various region types (non-coding, regulatory, coding) within the 246 known genetic variants provided by GWAS Catalog. We compared 399 reported and mapped genes to our list of DEGs. We then cross-referenced these genes with the PD gene online resource which analyzed over 800 publications and seven million polymorphisms (Lill et al., 2012). We selected five genes that remained significant in the PD gene meta-analysis, were represented in at least two studies, and had their most significant variant in a non-coding region (Table 2). We also selected three additional genes from the PD gene database that were represented in our RNA sequencing data (Table 2). The first, *UBOX5*, was among the most significant polymorphisms identified by the meta-analysis (Lill et al., 2012; Nalls et al., 2014). The other two, *PARK2* and *CHCHD2*, have significant polymorphisms according to the PD gene database but are also reported to have autosomal mutations that contribute to familial disease cases (Lill, 2016).

### Region Selection for Bisulfite and ChIP Primer Design

CCCTC-binding factor transcription factor binding was observed using the Uniform Transcription Factor Binding data found in the ENCODE Regulation super track in UCSC Genome Browser. We selected all CTCF transcription factor binding sites detected with ChIP-seq experiments from the ENCODE consortium from 2007 to 2012 (ENCODE Project Consortium, 2012). We also predicted which cytosine would overlap the binding motif using the CTCF binding prediction tool database v2.0 (Ziebarth et al., 2012). Primer design was focused on CTCF binding sites for both bisulfite sequencing and ChIP-qPCR experiments (further described below).

### Bisulfite-DNA Conversion and Bisulfite-Amplicon Sequencing Library Construction

Genomic DNA was extracted from two replicates of DMSO or rotenone-treated HEK293 using phenol: chloroform: isoamyl alcohol (Sigma, St. Louis, MO, United States). A total of 200 ng DNA was bisulfite-converted using the Sigma DNA Imprint Modification kit two-step protocol. Bisulfite-converted DNA (BS-DNA) was amplified with primers for selected regions designed with MethPrimer (Li and Dahiya, 2002) (Supplementary Table 2). Amplified BS-DNA products were run on a 2% EtBr agarose gel and purified using the mini-Elute gel extraction kit from Qiagen (Hilden, Germany). Purified products for each sample were pooled together, and 1 ng was used for library preparation using the Illumina Nextera XT DNA Library Preparation kit. Each sample was tagged with a unique Nextera XT adapter (San Diego, CA, United States). Sequencing libraries were quality checked via Bioanalyzer and run on an Illumina MiSeq platform to generate 150-bp paired-end reads.

### Bisulfite-Amplicon Sequencing Analysis for Methylation Patterns at CTCF Binding Sites

The raw fastq files were imported into the Galaxy web platform (Afgan et al., 2016). Reads with quality score <30 were filtered out, and reads with quality score >30 were trimmed with Trim Galore (Krueger, 2015). Reads were mapped to the human genome (hg19) using bwa-meth (Pedersen et al., 2014). MethylDackel was used for methylation calling, and per-cytosine contexts were merged into per-CPG metrics^1^. Duplicates and singletons identified in alignment were ignored from the methylation call. Minimum and maximum per-base depths were 1000× and 100,000×, respectively. The output was selected for methylKit format. Coverage statistics and differentially methylated regions were calculated for CG sites with methylKit installed in R (v3.5) (Akalin et al., 2012). Differentially methylated cytosines were defined as being present in both biological replicates, having a minimum absolute difference of 1% using the coverage weighted mean and having a SLIM adjusted *q*-value < 0.01 using the methylKit logistic regression model (Ning et al., 2011). The change in mean percent methylation (Δme) for all CpG sites within a defined region was calculated by taking the mean number of methylated versus non-methylated CpG sites from the pooled control and treated samples and using Fisher’s exact test FDR < 0.05.

### Chromatin Immunoprecipitation

All chemicals were purchased from Sigma unless otherwise noted (St. Louis, MO). HEK293 cells were harvested after a 24-h treatment and resuspended in fresh media at 10 × 10^6^ cells/mL in a conical tube. Cells were fixed with 1% formaldehyde for 10 min at room temperature. Reaction was stopped with 0.2 M glycine and incubation at room temperature for 5 min. Fixed cells were centrifuged for 5 min at 300 × g and 4°C and washed with 1 mL cold PBS. Fixed cell pellet was stored at -80°C until chromatin immunoprecipitation (ChIP).

Cell pellets were resuspended at approximately 1 × 10^6^ cells/0.1 mL with PBS + 0.5% Triton-X + 1% protease inhibitor cocktail and incubated on ice for 10 min prior to centrifugation for 5 min at 400 × g 4°C. The pellet was resuspended in TE buffer pH 8.0 with protease inhibitor and PMSF. Cells were sonicated at high intensity for 30 s on/60 s off until DNA fragments were within 200–800 bp as checked by 2% agarose gel. After sonication, samples were centrifuged for 15 min at 14,000 × g 4°C to pellet insoluble material. Sheared chromatin was transferred to RIPA buffer, and 10% of total chromatin was saved for input DNA extraction.

Chromatin immunoprecipitation was done with Dynabeads Protein A (Invitrogen, Carlsbad, CA, United States) and 4 μg of primary ChIP-grade antibody (H3K27ac Abcam ab4729; CTCF Millipore 07-729; Rabbit IgG Santa Cruz Biotechnology sc-2025). Beads were washed with lithium chloride (LiCl 0.25 M) buffer, and immunoprecipitated DNA was extracted from beads using the phenol: chloroform method. DNA was quantified using Qubit dsDNA high-sensitivity assay (Thermo Fisher Scientific, Waltham, MA, United States).

### ChIP-qPCR Analysis

We selected eight genes for quantitative real-time PCR (qPCR) analysis using primers listed in Supplementary Table 3. Primers were designed with NCBI Primer Blast at H3K27ac peaks surrounding the predicted CTCF binding site (Ye et al., 2012). All qPCR reactions were performed on a 7500 Real-Time PCR system from Applied Biosystems (Foster City, CA, United States) using the iTaq Universal SYBR Green Supermix from Bio-Rad (Hercules, CA, United States). H3K27ac and CTCF enrichment was calculated from the Ct threshold value as a percent of the total input DNA. Rabbit IgG samples were used as a negative control (Figures 5, 6B).

## Results

### Rotenone-Induced Stress Alters Transcription Factor Intracellular Signaling

**FIGURE 1:**
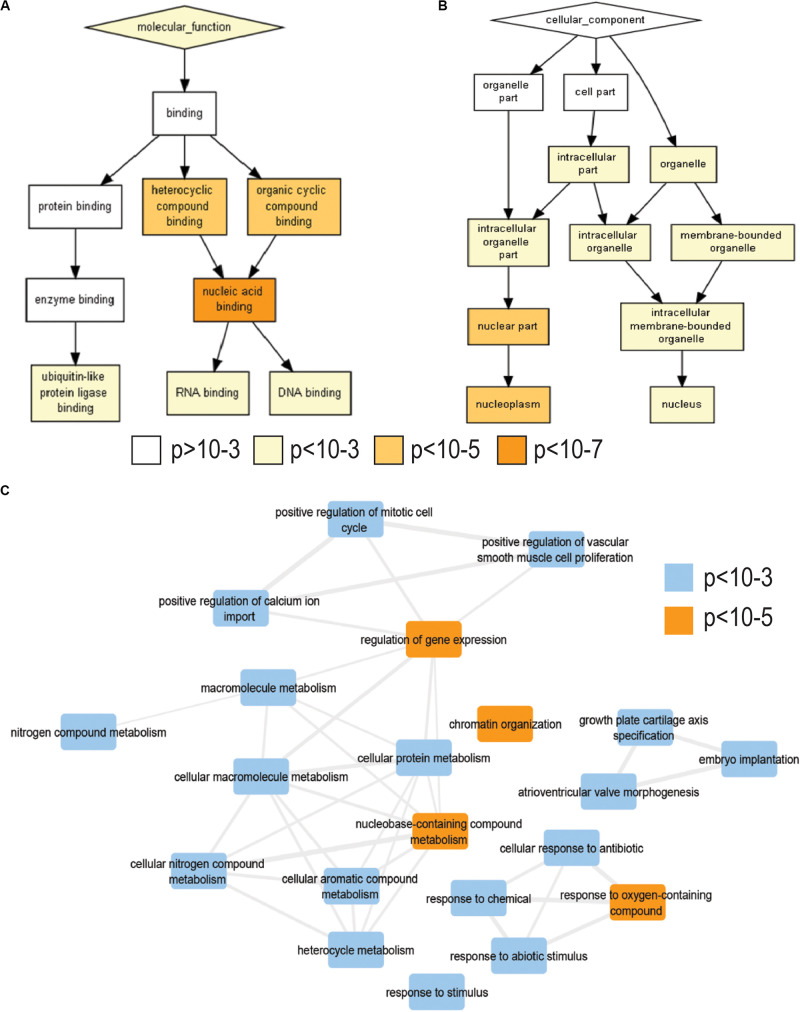
Gene Ontology enrichment analysis of RNA-sequencing data. **(A)** Gene Ontology molecular functions enriched in our differentially expressed genes from rotenone-treated HEK293 cells. **(B)** Gene Ontology cellular component enriched. Color gradient indicates enrichment *p*-value: white, >10^– 3^; yellow, 10^– 3^–10^– 5^; light orange, 10^– 5^–10^– 7^; dark orange, <10^– 7^. Enrichment *p*-value and adjusted FDR for each term are listed in Supplementary Tables 5, 6. **(C)** Network analysis of significantly enriched biological processes. Blue boxes enrichment *p*-value < 10^– 3^ and orange boxes enrichment *p*-value < 10^– 5^. Rotenone treatment changes the expression of genes involved in transcription factor signaling in the nucleus.

To characterize the changes in gene expression upon rotenone exposure, we treated HEK293 cells with rotenone 200 nM for 24 h. We then used RNA-seq analyses to identify over 2000 DEGs in response to rotenone (Supplementary File 3). To gain insights of impacted biological processes, we performed gene ontology enrichment analysis on 1853 of these DEGs with known HUGO (hgnc) symbol and cell description using a *p*-value threshold of *p* < 10^–3^. Among enriched biological processes, we observed a significant induction of the oxidative stress response, transcription factor activity, and chromatin organization (<10^–3^) (Figure 1C). We observed a significant enrichment of genes involved in nucleic acid binding (<10^–7^) and DNA binding (<10^–5^) with the nuclear cell component being most represented (<10^–5^) (Figures 1A,B and Supplementary Tables 5, 6). We analyzed pathway nine enrichment of the top 200 genes with the largest change in expression using reactome pathways (Fabregat et al., 2018; Supplementary Table 4). Three of the top five pathways enriched in our data were major transcription factor pathways including SMAD, NOTCH, and TP53 which have implications in PD reviewed in the discussion section.

### Alteration of PD-Associated Genes Stands Out Upon Rotenone Exposure

**FIGURE 2:**
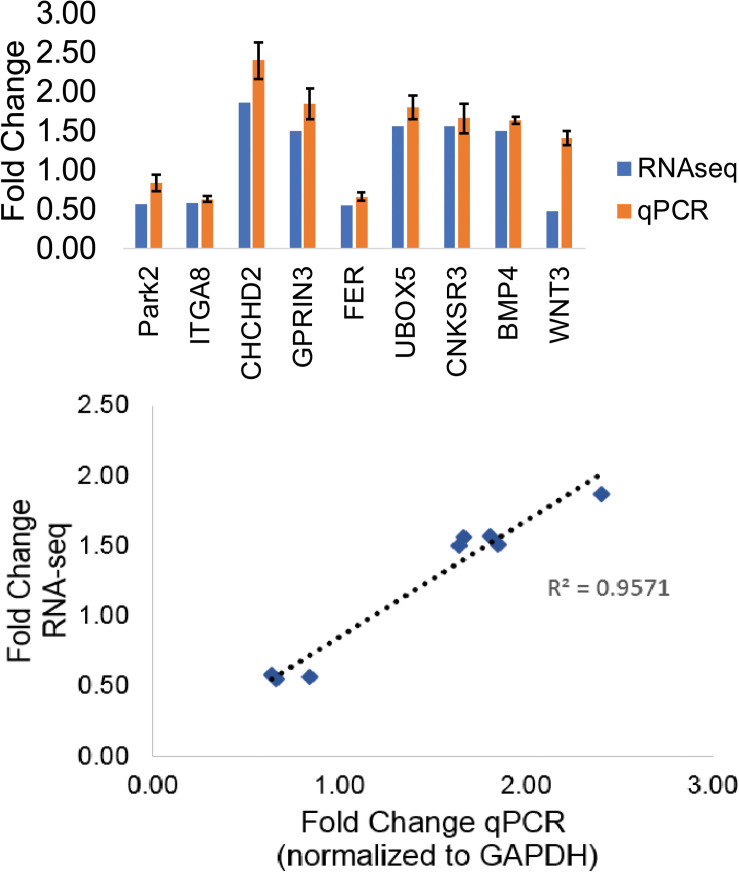
RNA-sequencing validation of PD-associated genes. Fold-change comparison of RNA sequencing results versus qPCR results and the linear calibration curve of RNA sequencing results with qPCR results expressed as fold change in expression.

The GWAS Catalog is a public database of approximately 72,000 variant–trait associations from over 3500 publications (Buniello et al., 2018). Out of 246 PD-associated variants with genetic sequence context information in the GWAS Catalog, 220 variants (89%) were in non-coding regions (intron, intergenic, regulatory, and exon) (Supplementary File 2). Intronic variants constituted most of the known polymorphisms. We searched our DEGs for PD-associated genes and identified 14 genes from the GWAS Catalog (Supplementary File 2). Of these genes, 13 were also considered significant PD-associated genes according to meta-analysis data in the PD gene (Lill et al., 2012). We validated the RNA sequencing results for 10 of these genes and were able to validate 8 of them with qPCR analysis (*R*^2^ = 0.96) (Figure 2). We selected five genes (*ITGA8*, *GPRIN3*, *FER*, *CNKSR3*, *BMP4*) and three additional genes from the PD gene meta-analysis (*UBOX5*, *PARK2*, *CHCHD2*) for further examination of epigenetic patterns (Table 1).

**TABLE 1:** Selected non-coding genetic variants.

HGNC	Log2 FC	FDR	Motif sequence	SNP*	Number of studies	*p*-value	Cell function
FER	−0.86	6.21E-07	AGCAGAGCA	rs13178668	13	<0.05	Tyrosine kinase activates cell surface signaling.
Park2	−0.82	3.55E-03	GTTGCCAGTAGGTGGCTCAC	rs4388272	13	<0.05	E3 ubiquitin ligase targets proteins for degradation.
ITGA8	−0.78	7.24E-06	GGAAGTCCA	rs10737104	15	2.70E-07	Transmembrane receptor activates various cell signaling.
BMP4	0.59	1.16E-02	GGAAGTGCG	rs148491084	3	<0.05	Secreted regulatory protein regulates development.
GPRIN3	0.60	3.31E-04	GGAACTGAA	rs10014765	15	6.48E-05	G-protein regulates neurite outgrowth.
CNKSR3	0.65	2.44E-05	CTCCCTCTACCTGT	rs145160741	12	<0.05	Scaffold protein signals membrane dynamics.
UBOX5	0.65	6.46E-04	CGTCCTCCAGTGGA	rs55785911	21	3.30E-10	Interacting protein signals ubiquitin proteasome pathway.
CHCHD2	0.90	2.00E-04	GGAAGAGCA	rs11978209	12	<0.05	DNAbinding protein signals oxidative stress response.

* SNP information found at PDGene Database (Lill et al., 2012)

**TABLE 2:** Regulomedb results for SNPs within selected regions.

Gene	chr:start	SNP ID	Rank	Score
*GPRIN3*	chr4:90228735	rs2116326	2a	0.96
*FER*	chr5:108084548	rs113728457	2a	0.92

### Selected Genes Contain Prominent CTCF Binding Sites in Their Regulatory Non-coding Regions

To examine any potential CTCF motifs within these selected genes, we visualized CTCF binding using experimental data from ENCODE and the CTCF binding prediction tool from the Cui Lab at the University of Tennessee (ENCODE Project Consortium, 2012; Ziebarth et al., 2012). Intriguingly, all selected genes had at least one prominent CTCF binding site in a regulatory non-coding region (Table 1). We designed both bisulfite primers and ChIP primers at these sites using ENCODE regulation data from the Broad Institute (ENCODE Project Consortium, 2012). The polymorphisms in these regions that were recognized by the SNP database (dbSNP^2^) were also analyzed with Regulomedb, a database that annotates SNPs with known or predicted interactions with regulatory elements in intergenic regions (Boyle et al., 2012). We determined that SNPs within selected regions at two genes, *GPRIN3* and *FER*, were present at CTCF binding sites and were active in the brain (Table 2). The rank of an SNP represents the number of available datasets for that polymorphism, and the score is generated based on the integrated results from available datasets. In this analysis, the polymorphism listed at each gene was present in datasets from experimental transcription factor binding, matched transcription factor position-weight matrix (PWM), and DNase footprinting. We checked the HEK293 genome using the online database^3^ and did not find either variant in our cells (Lin et al., 2014). This information provides additional evidence that CTCF binding sites among common non-coding variants may be critical in disease pathogenesis.

### Rotenone Modifies Epigenetic Patterns Across the Genome

**FIGURE 3:**
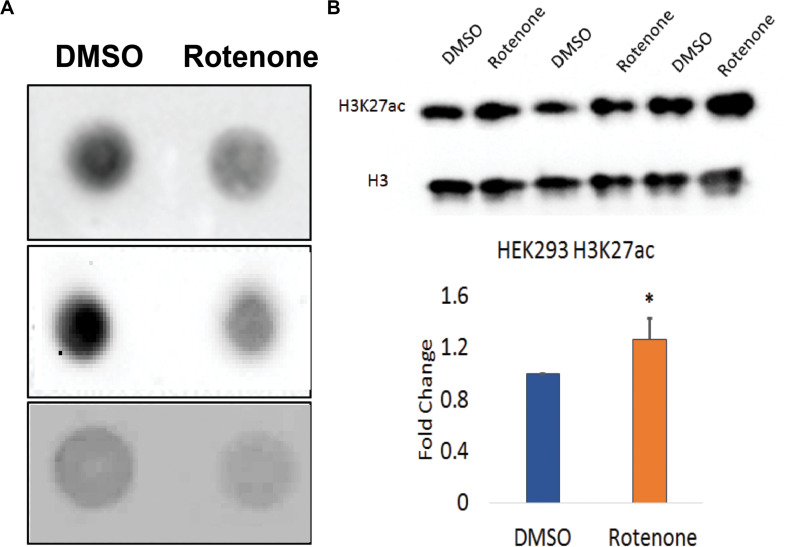
Global epigenetic patterns in rotenone treated *HEK293* cells. **(A)** DNA methylation was visualized by dot blot method using the anti-5mC antibody. Three biological replicates are shown in this image. The standard ladder using 100% 5mC standard is shown in Supplementary Figure 1. **(B)** Global histone H3K27ac levels were measured from total extracted histones using Western blot. Total histone H3 was used as the loading control. Three biological replicates are shown in this image. This western blot was quantified using ImageJ software and is shown as the fold change in the amount of H3K27ac relative to the vehicle (DMSO) control. ^∗^*p* < 0.05 using a paired Student’s *t*-test with the ratio of H3K27ac/H3.

We used the dot blot method to qualitatively assess changes in DNA methylation levels in cells exposed to rotenone. The total methylation level was detected with anti-5mC antibody and visualized using chemiluminescence. After 24 h, 5mC levels were strikingly reduced in genomic DNA (Figure 3A).

Next, we asked the extent to which rotenone exposure impacted histone acetylation. To investigate histone acetylation at active enhancers with PD-relevant SNPs, we selected H3K27ac mark (Wang et al., 2008) and examined H3K27ac levels from extracted histones using western blot. We measured a significant 1.3-fold increase in H3K27ac in rotenone-treated cells compared to the DMSO vehicle control (*p* < 0.05) (Figure 3B).

### Rotenone Alters DNA Methylation Patterns at CTCF Binding Sites in Regulatory Regions of PD-Associated Genes

**FIGURE 4:**
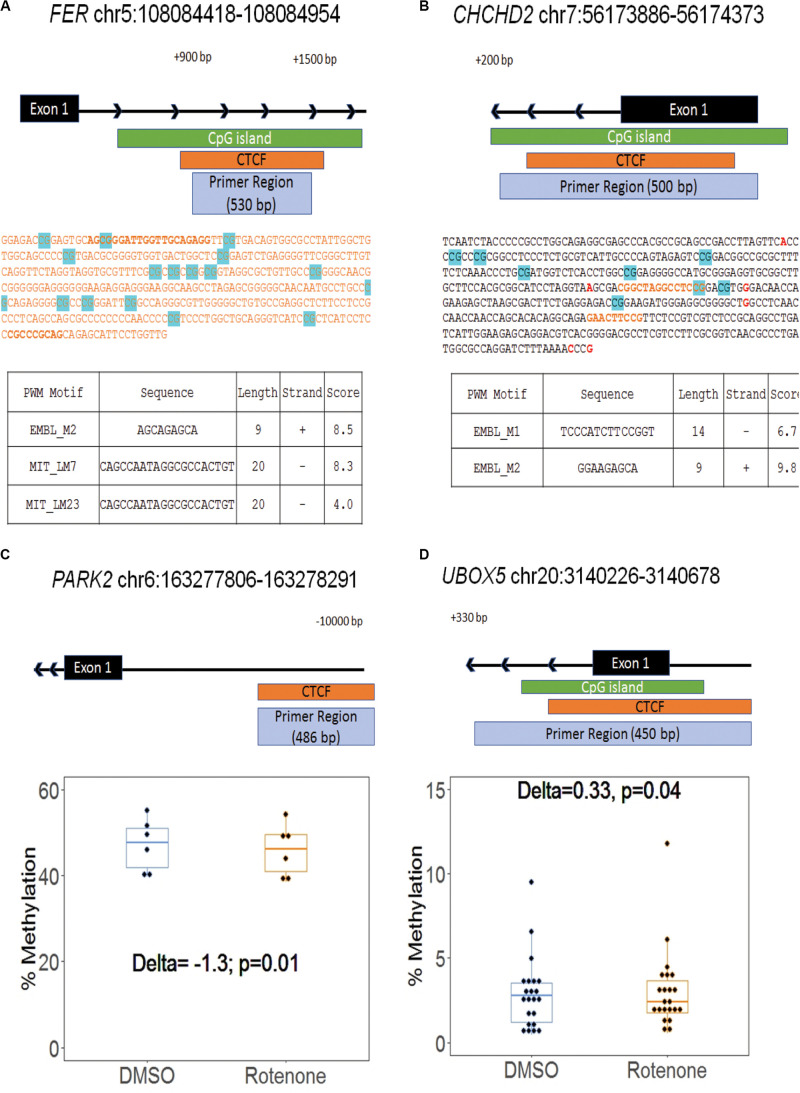
Differential methylation within CTCF motifs at Parkinson’s disease genes. **(A,B)** Two genes, *FER and CHCHD2*, had differential methylation at CG sites within their predicted CTCF binding motif. The amplified region at both genes covered the first exon and intron. The highlighted blue CG sites are all with significant differential methylation (>|1%|; *q*-value < 0.05). Red text emphasizes a common SNP. Orange text represents CTCF binding sites in the human genome identified by ENCODE. The bold orange text is the sequence motif predicted by the Cui Lab CTCF prediction tool (Ziebarth et al., 2012). The output of the CTCF binding prediction tool is listed in the table with the name of the position weight matrix motif, motif sequence, motif length, strand orientation, and the integrated output score. **(C,D)** Two genes, *PARK2* and *UBOX5*, had a significantly different methylation across the region. Each dot represents a CG within the region. Delta indicates the change in the mean CpG methylation percentage and the associated *p*-value from Fisher’s exact test.

Because CTCF binding is methylation sensitive and changes in CG methylation correlate with disease risks (Wang et al., 2012), we next examined a total of 284 CG nucleotides from eight regions surrounding our selected genes. Our amplicon-sequencing results demonstrated that 233 of these nucleotides met our minimum requirement of 1000 × coverage (Supplementary Figure 2). We focused our analysis on 165 CG sites that met minimum coverage requirements and overlapped predicted CTCF binding motifs at seven of the selected genes. There were 45 differentially methylated CG sites, and 53% were hypermethylated (Table 3). Two of these CG sites, *FER* cg143 at chr5:102025097 and CHCHD2 cg217 at chr7:56174103, were significantly hypomethylated (*FER cg143*Δ = −4.4) and hypermethylated (*CHCHD2 cg217*Δ = 1.7) at the predicted CTCF binding sequence (Figures 4A,B). The DNA sequence of the CTCF motif was predicted with the CTCF prediction tool by Ziebarth et al. (2012). The different motif sequences, or PWMs, found within the enhancer of these two genes are listed in Figures 4A,B. The score of each PWM corresponds to the log-odds of the observed sequence being specific rather than randomly generated. Two genes, *PARK2* and *UBOX5*, were significantly hypomethylated (*PARK2*Δ = −1.3) and hypermethylated (*UBOX5*Δ = 0.33) across the entire CTCF binding region with *p* < 0.05 but did not remain significant after multiple hypothesis testing (FDR > 0.05) (Figures 4C,D). Collectively, we conclude that methylation of CTCF binding motifs was vulnerable to rotenone exposure.

**FIGURE 5:**
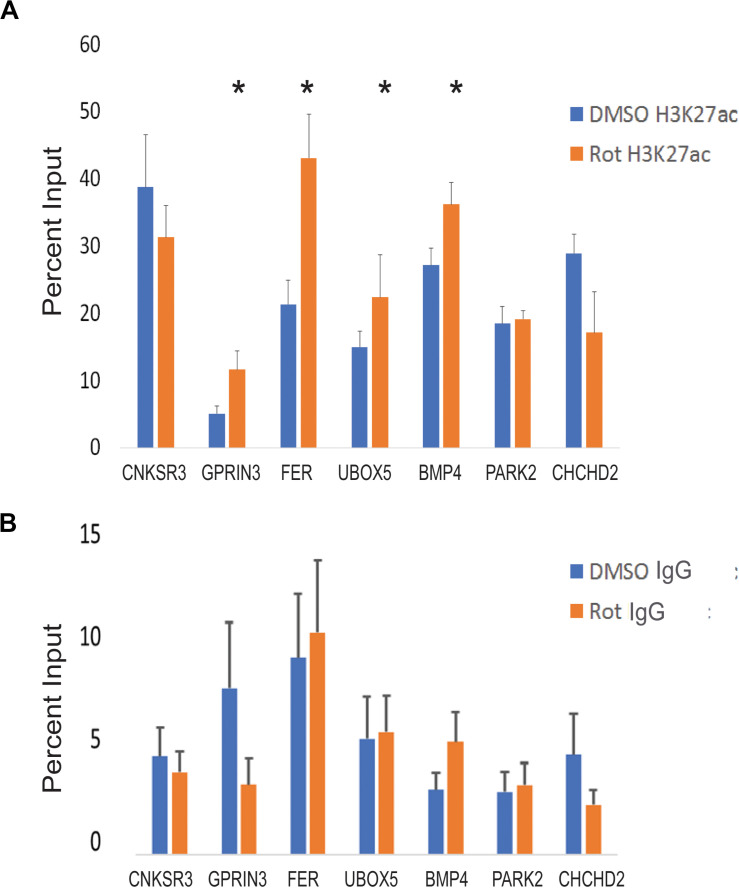
CTCF site H3K27 enhancer activation in response to rotenone. **(A)** The local abundance of H3K27ac within CTCF binding sites at Parkinson’s disease-associated genes was measured with ChIP-qPCR and expressed as the percent of total DNA input used for immunoprecipitation. **(B)** The negative control for ChIP analysis was Rabbit IgG. Significance was tested with paired Student’s *t*-test using the percent input of vehicle (DMSO) vs rotenone, and *post hoc* analysis for multiple hypotheses was done using the false discovery method. *FDR < 0.05.

**FIGURE 6:**
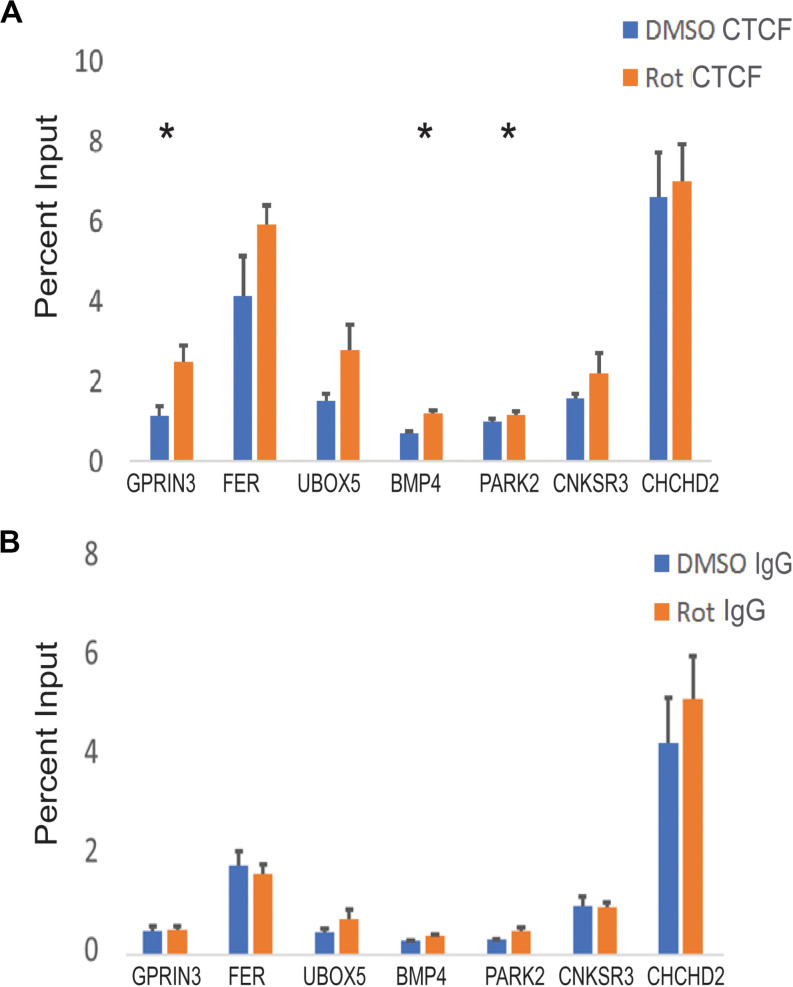
CTCF binding at PD-associated genes in response to rotenone. **(A)** The local abundance of CTCF binding at Parkinson’s disease-associated genes was measured with ChIP-qPCR and expressed as the percent of total DNA input used for immunoprecipitation. **(B)** The negative control for ChIP analysis was Rabbit IgG. Significance was tested with paired student’s *t*-test using the percent input of vehicle (DMSO) vs rotenone and *post hoc* analysis for multiple hypotheses was done using the false discovery method. *FDR < 0.05.

**TABLE 3:** Differentially methylated CG sites at Parkinson’s disease-associated genes.

CHR_GENE	CG	*p*-value	*q*-value	Delta
chr14_54422869_54423420_BMP4	54423352	5.48E-08	5.60E-08	−3.3
chr20_3140226_3140678_UBOX5	3140420	2.59E-27	9.41E-27	2.3
chr20_3140226_3140678_UBOX5	3140429	3.46E-34	1.62E-33	3.6
chr4_90228647_90229070_GPRIN3	90228692	2.37E-07	2.24E-07	−1.1
chr4_90228647_90229070_GPRIN3	90228700	1.87E-13	3.06E-13	1.3
chr4_90228647_90229070_GPRIN3	90228702	3.42E-13	5.41E-13	1.2
chr4_90228647_90229070_GPRIN3	90228709	1.23E-33	5.49E-33	−2.5
chr4_90228647_90229070_GPRIN3	90228753	2.38E-09	2.82E-09	1.1
chr4_90228647_90229070_GPRIN3	90228761	1.33E-13	2.29E-13	1.7
chr4_90228647_90229070_GPRIN3	90228764	4.10E-07	3.73E-07	1.0
chr4_90228647_90229070_GPRIN3	90228792	2.58E-11	3.61E-11	−1.2
chr4_90228647_90229070_GPRIN3	90228822	4.03E-16	8.78E-16	1.7
chr4_90228647_90229070_GPRIN3	90228849	1.47E-36	8.03E-36	2.7
chr4_90228647_90229070_GPRIN3	90228860	3.23E-44	2.88E-43	1.8
chr5_108084418_108084954_FER	102025087	1.53E-06	1.26E-06	−1.6
chr5_108084418_108084954_FER	102025097	5.23E-76	8.56E-75	−4.4
chr5_108084418_108084954_FER	102025117	1.62E-140	1.59E-138	−4.9
chr5_108084418_108084954_FER	102025151	2.40E-14	4.62E-14	−1.3
chr5_108084418_108084954_FER	102025176	2.79E-12	4.14E-12	1.6
chr5_108084418_108084954_FER	102025225	1.05E-16	2.39E-16	1.9
chr5_108084418_108084954_FER	102025228	1.12E-19	2.98E-19	3.4
chr5_108084418_108084954_FER	102025231	1.23E-11	1.79E-11	−2.8
chr5_108084418_108084954_FER	102025234	1.56E-15	3.26E-15	1.7
chr5_108084418_108084954_FER	102025251	4.73E-03	2.52E-03	−1.8
chr5_108084418_108084954_FER	102025320	5.38E-07	4.77E-07	1.9
chr5_108084418_108084954_FER	102025330	3.76E-04	2.32E-04	1.3
chr5_108084418_108084954_FER	102025334	2.60E-04	1.67E-04	−1.7
chr5_108084418_108084954_FER	102025341	1.36E-15	2.90E-15	2.7
chr5_108084418_108084954_FER	102025408	2.37E-07	2.24E-07	2.1
chr5_108084418_108084954_FER	102025430	2.26E-03	1.28E-03	−1.4
chr6_154830537_154830958_CNKSR3	154830543	8.22E-22	2.44E-21	−2.5
chr6_154830537_154830958_CNKSR3	154830584	1.66E-24	5.63E-24	1.8
chr6_154830537_154830958_CNKSR3	154830770	2.43E-59	2.65E-58	−1.5
chr6_154830537_154830958_CNKSR3	154830810	1.02E-43	8.35E-43	1.7
chr6_154830537_154830958_CNKSR3	154830817	3.60E-14	6.66E-14	−1.1
chr6_154830537_154830958_CNKSR3	154830837	1.41E-93	6.93E-92	3.3
chr6_154830537_154830958_CNKSR3	154830863	8.54E-20	2.33E-19	−1.0
chr6_163277806_163278291_PARK2	163277841	3.89E-04	2.38E-04	−1.7
chr6_163277806_163278291_PARK2	163277943	8.27E-07	7.12E-07	−2.0
chr7_56173886_56174373_CHCHD2	56174016	1.67E-08	1.76E-08	−1.1
chr7_56173886_56174373_CHCHD2	56174033	4.52E-04	2.75E-04	−1.9
chr7_56173886_56174373_CHCHD2	56174103	1.71E-05	1.29E-05	1.7
chr7_56173886_56174373_CHCHD2	56174107	1.01E-08	1.11E-08	−1.1
chr7_56173886_56174373_CHCHD2	56174149	2.07E-04	1.37E-04	1.6
chr7_56173886_56174373_CHCHD2	56174179	3.24E-04	2.05E-04	1.2

### Rotenone Alters H3K27ac at CTCF Binding Sites in Regulatory Regions of PD-Associated Genes

We used ChIP-qPCR to test whether local H3K27ac enrichment overlapped CTCF binding sites in PD-associated genes. Our ChIP-qPCR results demonstrate that four genes (*GPRIN3*, *UBOX5*, *FER*, and *BMP4*) had significantly increased H3K27ac at CTCF binding motifs with FDR < 0.05. One gene, *CNKSR3*, had reduced H3K27ac at its CTCF binding motif but was not statistically significant (*p* = 0.07; FDR = 1) (Figure 5A). H3K27ac enhancer activity was correlated with gene upregulation in three genes (*GPRIN3, UBOX5, BMP4*). *FER* was downregulated despite increased H3K27ac in its first intron but may be related to changes in DNA methylation within that region, altered posttranscriptional regulation, or limitations of targeted ChIP analysis. Interestingly, the H3K27 region amplified in qPCR overlapped at least one differentially methylated cytosine for all four significantly enhanced genes (Table 4). Only one of the genes without H3K27ac enrichment, *CHCHD2*, also had differentially methylated cytosines within the amplified region. These genes had both increased and decreased changes in percent methylation.

**TABLE 4:** Differentially methylated CG sites within H3K27ac-enriched regions.

CHR_GENE	CG	*p*-value	*q*-value	Delta
chr14_54422869_54423420_BMP4	54423352	5.48E-08	5.60E-08	−3.3
chr20_3140226_3140678_UBOX5	3140420	2.59E-27	9.41E-27	2.3
chr20_3140226_3140678_UBOX5	3140429	3.46E-34	1.62E-33	3.6
chr4_90228647_90229070_GPRIN3	90228822	4.03E-16	8.78E-16	1.7
chr4_90228647_90229070_GPRIN3	90228849	1.47E-36	8.03E-36	2.7
chr4_90228647_90229070_GPRIN3	90228860	3.23E-44	2.88E-43	1.8
chr5_108084418_108084954_FER	1.02E + 08	1.05E-16	2.39E-16	1.9
chr5_108084418_108084954_FER	1.02E + 08	1.12E-19	2.98E-19	3.4
chr5_108084418_108084954_FER	1.02E + 08	1.23E-11	1.79E-11	−2.8
chr5_108084418_108084954_FER	1.02E + 08	1.56E-15	3.26E-15	1.7
chr5_108084418_108084954_FER	1.02E + 08	4.73E-03	2.52E-03	−1.8
chr5_108084418_108084954_FER	1.02E + 08	5.38E-07	4.77E-07	1.9
chr5_108084418_108084954_FER	1.02E + 08	3.76E-04	2.32E-04	1.3
chr5_108084418_108084954_FER	1.02E + 08	2.60E-04	1.67E-04	−1.7
chr5_108084418_108084954_FER	1.02E + 08	1.36E-15	2.90E-15	2.7
chr5_108084418_108084954_FER	1.02E + 08	2.37E-07	2.24E-07	2.1
chr5_108084418_108084954_FER	1.02E + 08	2.26E-03	1.28E-03	−1.4

### Rotenone Increases CTCF Binding at Three PD-Associated Genes

To determine whether altered DNA methylation and H3K27ac patterns would affect CTCF binding, we measured CTCF enrichment at its binding motif at seven PD-associated genetic loci. CTCF binding was increased at three genes (*PARK2, GPRIN3*, and *BMP4*) (Figure 6A). *BMP4* had one hypomethylated CG and increased H3K27ac within our selected region. There was an increase in CTCF binding and mRNA expression. *PARK2* had two hypomethylated CGs but no increase in H3K27ac in its CTCF binding domain. In this region, CTCF binding increased and mRNA expression decreased. *GPRIN3*, unlike the other two genes, had more hypermethylated CGs within its CTCF binding motif but the closest CG to its consensus sequence was also hypomethylated. There was increased H3K27ac enrichment at *GPRIN3* and increased CTCF binding. *GPRIN3* mRNA was significantly upregulated in response to rotenone. These data suggest that both DNA methylation and H3K27ac influence CTCF transcription factor binding and impact the expression of PD-associated genes (Table 5).

**TABLE 5:** Correlation of gene expression changes with CTCF binding at PD-associated genes.

	*PARK2*	*GPRIN3*	*BMP4*
RNA	↓	↑	↑
Methylation	↓	↑↓	↓
H3K27ac	–	↑	↑
CTCF	↑	↑	↑

## Discussion

We selected rotenone based on its ability to model gene–environment interactions in rodents and non-mammalian models of PD (Cannon and Greenamyre, 2013; Johnson and Bobrovskaya, 2015). It is estimated that chronic exposure to concentrations of approximately 20–30 nM of rotenone is enough to cause degeneration of dopaminergic neurons in the midbrain (Greenamyre et al., 2003). While rotenone is a potent inhibitor of complex I in the electron transport chain of mitochondria, rotenone has been shown to cause neurodegeneration by mechanisms unrelated to its effect on complex I (Sherer et al., 2007; Choi et al., 2008). The transcriptome and its regulation have become a focus for understanding these mechanisms outside of the electron transport chain (Cabeza-Arvelaiz and Schiestl, 2012). We observed large-scale changes in gene expression profiles, and many of these genes were enriched in processes involved in gene regulation and chromatin organization (Figure 1). The pathway analysis of DEGs also revealed a large involvement in major intracellular transcription factor pathways. For instance, SMAD proteins are critical for transducing signals from the transforming growth factor (TGFβ) receptors at the plasma membrane which are essential for midbrain dopaminergic survival (Hegarty et al., 2014). Notch signaling is known to have an important role in regulating genes involved in nervous system development and synaptic plasticity (Ables et al., 2011). Lastly, the TP53 pathway is perhaps the most well-known of the toxicant-induced signaling mechanisms to control cell cycle progression and cellular survival. It is thus a critical regulator of programmed cell death in PD and rotenone-induced neurotoxicity (Venderova and Park, 2012).

To determine if the gene expression changes discussed above were due to changes in global levels of epigenetic patterns, we performed dot blots and Western blots to examine global levels of DNA methylation (5mC) and H3K27ac (Figures 3A,B). DNA methylation is the best-studied epigenetic modification, and small changes in methylation at regulatory regions of the genome can have substantial effects on genome integrity during aging (Horvath, 2013). As seen with other pesticide models, we observed a global decrease in total 5mC in response to rotenone. We also chose to look at global H3K27ac levels because it is tightly correlated with gene expression and vulnerable to environmentally driven enhancer activation (Wang et al., 2008). We observed a significant increase in H3K27ac across the genome. H3K27ac is not only an important mark to distinguish poised from active enhancers in bivalent chromatin but also a critical epigenetic modulator in post-mitotic neurons (Maze et al., 2015).

We searched the DEGs for non-coding risk variants associated with PD (fc = fold change). We discovered 13 genes with significant association to PD using GWAS meta-data. We focused on eight genes (*ITGA8* 0.6 fc, *GPRIN3* 1.5 fc, *FER* 0.6 fc, *CNKSR3* 1.6 fc, *BMP4* 1.5 fc, *UBOX5* 1.6 fc, *PARK2* 0.6 fc, and *CHCHD2* 1.9 fc) that remained significantly associated in at least two studies with their most significant variant lying in a non-coding region (PDGene; Lill et al., 2012). *UBOX5* was the most significantly associated variant according to GWAS meta-data. Furthermore, *UBOX5* was the only identified gene with its most significant non-coding variant having known interactions with regulatory elements such as CTCF (Regulomedb; Boyle et al., 2012).

*UBOX5* is predicted to have a role in the ubiquitin proteasome system, a well-known PD pathway involved in protein quality control and cellular detoxification (McNaught and Jenner, 2001; McNaught et al., 2003). This pathway is involved in the function of multiple PD-associated genes most notably *PARK2* which encodes a ubiquitin ligase. Mutations in *PARK2* account for approximately 50% of familial early-onset PD, but the frequency of these mutations decreases with age (Bekris et al., 2010). These mutations generally occur at exon sequences, but other less penetrant but significantly associated polymorphisms with higher frequency in the population occur more often at intronic or regulatory sequences of the gene.

Each of the selected genes had a CTCF binding site determined by ENCODE and the CTCF prediction tool (ENCODE Project Consortium, 2012; Ziebarth et al., 2012). We used the online tool, Regulomedb, to investigate whether SNPs in these CTCF binding regions had evidence of an interaction with CTCF (Table 2). There was evidence of a CTCF interaction in the brain in two of the selected genes *GPRIN3* and *FER*. This rank score generated by Regulomedb is indicative of the strength of the evidence for this interaction with one being the greatest.

CCCTC-binding factor binds regions with allele-specific methylation and preferentially binds the unmethylated allele (Wang et al., 2018). The methylation status of these allele-specific methylated regions is critical to functional CTCF binding and can explain as much as 41% of its variability (Wang et al., 2012). We have previously identified allele-specific methylated regions in the human genome and verified their sensitivity to rotenone exposure (Martos et al., 2017; Freeman et al., 2020). Therefore, we hypothesized that CTCF binding sites at PD-associated genes would also be vulnerable to rotenone. Out of 165 CG sites that met minimum coverage requirements and overlapped predicted CTCF binding motifs, we detected 45 differentially methylated cytosines (Table 3). In two of the genes, the cytosines within the CTCF consensus sequence were differentially methylated but not in any consistent direction (*FER-*hypomethylated; *CHCHD2*-hypermethylated) (Figures 4A,B). We saw a similar trend in *PARK2* and *UBOX5* which had differential methylation across the whole binding region but did not change in a consistent direction (*PARK2*-hypomethylated) and (*UBOX5*-hypermethylated) (Figures 4C,D). Overall, there was a slight increase in hypermethylated cytosines (53%) indicating a potential decrease in CTCF binding capacity.

One of the primary functions of CTCF is to act as an insulator by blocking enhancer–promoter interactions (Phillips and Corces, 2009). CTCF is thus tightly correlated with enhancer activity, and its interaction with active enhancers topologically is much greater than with silent regions of the genome (Ren et al., 2017). Histone acetylation patterns also determine chromatin structure, and the histone mark H3K27ac is correlated with active enhancer regions (Wang et al., 2008; Creyghton et al., 2010). p300 is one of the primary histone acetyltransferase enzymes and loads the acetyl group onto the lysine tail of histone 3 at active regions. CTCF binding sites are often located next to at least one p300 binding site and interacts with p300 at the chromatin with active acetylation (Ren et al., 2017).

Histone acetylation patterns are vulnerable to environmental factors and like DNA methylation are heritable (Chinnusamy and Zhu, 2009; Dai and Wang, 2014; Zhu et al., 2018). It is likely that histone acetylation patterns also contribute to the role of genetic variants in disease pathogenesis. We tested H3K27ac levels at CTCF binding sites to determine if acetylation patterns were also sensitive to rotenone at PD-associated genes. We saw an increase in H3K27ac at four of the eight identified genes suggesting strong chromatin interactions with CTCF (FDR < 0.05) (Figure 5A). Notably, all four genes with H3K27ac also overlapped a differentially methylated cytosine within the CTCF binding region (Table 4). This suggests a cross talk mechanism with DNA methylation patterns and H3K27ac enrichment at CTCF binding sites to control chromosomal organization and SNP impacted gene expression.

We observed increased CTCF binding at three differentially expressed PD-associated genes (Figure 6A). *PARK2* is a well-known genetic factor in PD as described earlier. Increased CTCF binding at its upstream enhancer decreased its expression, thereby affecting its role in the ubiquitin proteasome system. *BMP4* is a gene that encodes bone morphogenetic protein 4, and it regulates neurite outgrowth and axonal transport through the activation of the TGFβ/Smad pathway which is disrupted according to RNA sequencing reactome enrichment data (Supplementary Table 4). Increased CTCF binding was associated with increased *BMP4* promoter expression which is essential for dopaminergic neuron differentiation and survival (Table 5; Hegarty et al., 2014). *GPRIN3* encodes a protein involved in microtubule dynamics and neurite outgrowth which are both impaired in rotenone-induced neurotoxicity (Cabeza-Arvelaiz and Schiestl, 2012). Interestingly, increased CTCF binding occurred at *GPRIN3* within an active transcription start site in the substantia nigra (Table 5; Boyle et al., 2012). It is also associated with a fully penetrant PD mutation causing a triplication of these loci and doubling of *GPRIN3* mRNA transcripts (Devine et al., 2011). This observed increase in mRNA transcripts in a mature dopaminergic neuron differentiated from a patient was comparable to the observed 1.5-fold change increase in our rotenone-treated cells.

## Data Availability Statement

RNA sequencing data has been submitted to the Gene Expression Omnibus (GEO) repository at NCBI Databank with record number GSE147617. Bisulfite-amplicon sequencing data is publicly available on the sequence read archive (SRA) at NCBI Databank with Bioproject ID PRJNA615220. All other data used to generate this report are available upon request from the corresponding author ZW (zwang47@jhu.edu).

## Author Contributions

DF designed and performed experiments with supervision from ZW. Both authors analyzed and interpreted data and prepared the manuscript.

## Conflict of Interest

The authors declare that the research was conducted in the absence of any commercial or financial relationships that could be construed as a potential conflict of interest.

## Footnotes

1 https://github.com/dpryan79/MethylDackel

2 http://www.ncbi.nlm.nih.gov/SNP

3 http://hek293genome.org/v2/
