# Supplementary material for: Epigenetic Vulnerability of Insulator CTCF Motifs at Parkinson’s Disease-Associated Genes in Response to Neurotoxicant Rotenone
Source: Front Genet. 2020 Jul 7;11:627. doi: 10.3389/fgene.2020.00627 (PMC7381335; doi:10.3389/fgene.2020.00627)
Supplement: Supplementary file 2 [file Data_Sheet_2.PDF]

Supplemental File 3: Differentially expressed genes from rotenone treated HEK293

Genename	logFC	FDR
STC1	-3.87	2.3E-50
PPP1R10	-1.88	4.1E-40
RN7SL1	-2.15	9.5E-37
ADAMTS1	1.76	1.7E-35
SH3BP5-AS1	-2.06	3.7E-34
NCOA5	-2.01	1.2E-31
ZNF37A	-1.71	2.1E-30
MT-TQ	2.97	1.8E-29
CYR61	1.88	7.4E-26
PCF11	-1.54	1.6E-25
ZNF605	-1.60	4.4E-25
KRCC1	-2.00	7.2E-25
MT-TP	2.82	7.6E-25
SAT1	1.79	5.3E-24
RN7SL2	-1.88	5.8E-24
HIST2H2BE	2.91	5.8E-24
ZSWIM5	-1.67	3.6E-23
TSC22D3	1.47	2.4E-22
FOSB	1.86	1.3E-21
ALMS1-IT1	2.54	3.2E-21
RIPK1	-1.93	7.0E-21
MEX3B	1.65	7.8E-21
THAP9-AS1	1.53	5.7E-20
ILF3-AS1	1.65	2.4E-19
PFKFB4	-1.85	3.6E-19
BRD2	1.35	4.7E-19
SOX4	1.19	7.9E-19
C1QTNF6	-1.68	1.0E-18
JUN	1.42	1.6E-18
PIM1	1.35	1.7E-18
ZNF280C	1.25	2.4E-18
RN7SK	-4.17	4.1E-18
ZNF587B	-1.78	9.9E-18
ARC	1.87	1.8E-17
HSPA5	1.17	2.4E-17
MSMO1	1.58	3.5E-17
TGIF1	1.41	6.2E-17
DOK3	-2.57	1.1E-16
PAQR6	-1.56	1.6E-16
MT-TA	2.80	2.1E-16
WDR52	-1.87	7.4E-16
B3GALT6	-1.47	9.3E-16
PPP1R18	1.20	1.0E-15

GAB2	1.67	1.7E-15
LRRC37A9P	-1.84	3.0E-15
CAPN12	1.78	1.7E-14
IQCE	-1.12	2.2E-14
SAMD9	-1.83	2.3E-14
CYP2U1	-1.61	2.4E-14
FZD6	-1.29	3.0E-14
ZBED3	-1.12	4.3E-14
PER1	1.37	6.7E-14
LGR5	-1.14	1.0E-13
C15orf38	-1.17	1.1E-13
ZC3H4	-1.48	1.3E-13
MYB	1.53	1.8E-13
INSIG1	1.24	2.0E-13
TUFM	1.05	2.0E-13
TSC22D2	1.10	2.0E-13
PPFIA4	-1.37	2.6E-13
PPP1R32	1.95	3.1E-13
TAF1D	1.14	3.9E-13
PRKACB	-1.19	4.3E-13
MID1IP1	1.29	4.6E-13
SDHAP3	-1.33	6.0E-13
LINC00680	-1.59	1.4E-12
NUTM2D	-1.90	1.6E-12
AK4	-1.04	2.8E-12
LOX	-1.87	3.8E-12
SLC39A3	1.57	4.1E-12
KCNAB3	-2.35	4.2E-12
BBC3	1.50	4.4E-12
FAM46B	2.42	4.4E-12
CNN2	1.15	5.1E-12
WNK4	-1.62	5.1E-12
PGK1	-1.08	5.1E-12
LRRC37A4P	-1.19	6.3E-12
RBM15	1.00	7.6E-12
FKRP	-1.19	1.1E-11
MT-ND2	-0.97	1.1E-11
HYAL3	-2.19	1.2E-11
C12orf4	-1.12	1.3E-11
LCMT2	-1.39	1.5E-11
SULF1	-1.18	1.5E-11
DNAJA1	-1.01	1.7E-11
HMGCS1	1.01	2.2E-11
GPR75	-1.54	2.2E-11
SQLE	0.94	2.6E-11
ATF3	1.24	3.9E-11
RPL18A	0.95	4.6E-11

FOXD2-AS1	1.28	4.6E-11
RHOB	1.14	4.8E-11
MAX	-0.98	4.9E-11
H1F0	1.07	5.4E-11
C11orf95	-1.03	5.6E-11
P2RY11	-1.70	5.7E-11
CSRNP1	1.44	6.2E-11
MYC	1.03	6.3E-11
P4HA1	-1.32	7.5E-11
EXO5	-1.22	1.0E-10
ADAM1A	-1.86	1.1E-10
SCRN3	-1.05	1.2E-10
LDLR	0.97	1.2E-10
FAM212B	-1.27	1.5E-10
ZNF302	-0.95	1.5E-10
PRNP	-0.91	1.7E-10
SLC16A13	-1.32	1.8E-10
GRB7	1.29	1.8E-10
CHAMP1	-0.96	1.9E-10
MT-CYB	-0.85	2.0E-10
ANKRD29	-1.25	2.1E-10
ZNF37BP	-0.91	2.1E-10
LRIF1	-1.14	2.2E-10
FAM178A	-1.02	2.2E-10
JUNB	1.63	2.3E-10
GNRHR2	2.62	2.4E-10
LINC00863	-1.47	2.5E-10
SFPQ	-0.88	2.5E-10
SERTAD3	1.19	2.6E-10
RNF168	1.01	2.8E-10
IKBIP	-1.25	2.8E-10
SLC16A9	-1.43	2.8E-10
ZNF449	0.97	3.0E-10
ID3	1.32	3.2E-10
TUFT1	1.00	3.7E-10
PSKH1	-1.27	4.8E-10
GBE1	-1.21	4.8E-10
ZNF526	1.01	4.8E-10
PARP9	-1.39	5.5E-10
PRIMPOL	-1.22	5.8E-10
BNIP3	-1.22	5.8E-10
RBM12	-0.94	6.3E-10
DUSP1	1.08	6.3E-10
ZNF252P	-1.45	6.7E-10
TMEM45A	-1.20	6.7E-10
ZNF333	1.03	6.9E-10
RPL13AP5	2.61	7.4E-10

ACTG1	0.88	7.5E-10
EMILIN3	-1.18	8.3E-10
ZNF467	1.71	8.8E-10
SRD5A3	-1.08	9.6E-10
PDE3B	-1.21	9.7E-10
SACS	-0.93	1.1E-09
KLF9	0.92	1.1E-09
CTGF	1.35	1.1E-09
OVGP1	1.26	1.2E-09
PLOD2	-1.19	1.2E-09
C22orf46	-1.07	1.3E-09
IER2	0.97	1.4E-09
TINCR	1.82	1.5E-09
PDP2	0.97	1.5E-09
HFM1	-1.46	1.7E-09
SRR	-1.20	1.7E-09
NREP	-0.94	1.8E-09
LPAR1	-1.12	1.8E-09
ZFX	-0.90	1.9E-09
MIR210HG	-3.61	2.1E-09
EPC2	-0.99	2.1E-09
MTND2P28	-0.93	2.3E-09
BRPF3	-0.91	2.4E-09
METTL7B	-1.55	2.7E-09
HELB	1.35	2.9E-09
C1orf63	0.91	3.1E-09
NR4A2	1.68	3.1E-09
ASMTL	1.18	3.3E-09
MYH3	-1.62	3.3E-09
JAG1	0.90	3.4E-09
SLC25A29	0.93	3.5E-09
HNRNPL	0.80	3.8E-09
NIPAL2	-1.61	4.5E-09
NEK9	-0.83	4.7E-09
QRICH2	-2.31	4.8E-09
FANCE	1.02	4.8E-09
GFOD2	1.02	5.0E-09
FAM131A	1.18	5.2E-09
METTL22	0.91	5.2E-09
MNX1	1.05	5.7E-09
DPY19L2P2	-1.48	5.7E-09
FLNA	0.85	7.0E-09
ARHGAP8	1.54	7.2E-09
CHTF8	-0.93	7.2E-09
FAM134B	-1.58	7.7E-09
MAST3	0.99	8.2E-09
HGSNAT	-0.92	9.1E-09

ZC3H12C	-1.41	9.2E-09
GLUL	0.78	9.7E-09
SRSF6	-0.80	9.9E-09
MMP15	1.10	1.1E-08
ZNF555	1.19	1.1E-08
MUC19	-1.32	1.2E-08
ZNF74	0.97	1.3E-08
ZNF75A	-0.95	1.3E-08
TRPM4	1.11	1.3E-08
ZNF883	-1.22	1.3E-08
KIAA1683	-2.92	1.4E-08
STAT4	-1.45	1.5E-08
SH3D21	-1.25	1.5E-08
CDKN2B	1.27	1.5E-08
SLC29A2	0.88	1.6E-08
WNT3	-1.06	1.6E-08
ZNF551	-1.27	1.7E-08
AMPD3	-1.92	1.8E-08
CDT1	1.01	1.9E-08
ZBTB43	0.95	2.0E-08
KCTD15	-0.84	2.3E-08
CDK13	-0.86	2.5E-08
CSPG4P12	-1.06	2.6E-08
SLC1A4	-1.36	2.7E-08
TBX19	1.53	2.8E-08
HOTAIRM1	0.97	2.8E-08
ZNF497	-2.32	3.0E-08
MFAP3	-0.96	3.0E-08
NAA10	1.00	3.0E-08
IGSF10	-1.21	3.7E-08
DGCR5	-1.12	4.3E-08
EXOC8	0.86	4.3E-08
SH2D3A	1.04	4.3E-08
TEX15	-0.98	4.3E-08
RPL17	1.03	4.3E-08
AGPAT3	-0.83	4.7E-08
SPOCK2	-1.72	4.7E-08
EIF3I	0.78	4.7E-08
MIER2	0.87	4.9E-08
MVP	1.29	5.0E-08
DDIT3	1.05	5.0E-08
RPPH1	-3.28	5.1E-08
YJEFN3	-1.12	5.3E-08
MCM3AP-AS1	-1.08	5.4E-08
DCP1B	-1.05	5.4E-08
ZBTB1	-1.02	5.7E-08
PSPC1	-0.80	6.1E-08

AURKC	1.45	6.1E-08
RNF122	1.09	6.2E-08
SALL4	1.82	6.2E-08
EIF5A	0.98	6.3E-08
ZNF235	-1.24	6.4E-08
JAK3	1.68	6.4E-08
RBBP6	-0.91	6.4E-08
KIAA1161	0.94	6.6E-08
RP1L1	1.83	7.0E-08
DDB2	-0.81	7.1E-08
GCAT	1.09	7.1E-08
FAM84B	0.76	7.4E-08
BNIP3L	-1.08	7.4E-08
PIEZO2	-1.13	7.5E-08
IL12A	1.27	7.5E-08
DRAP1	0.93	8.1E-08
SRSF3	-0.76	8.4E-08
ICAM5	1.00	8.4E-08
LDHA	-0.74	8.5E-08
NR1D1	1.24	8.5E-08
TRAK2	-0.86	8.6E-08
ZDHHC22	1.05	8.6E-08
ZNF585B	-0.89	8.6E-08
CCDC124	0.82	8.9E-08
ZNF594	-1.34	8.9E-08
ALX4	-1.01	9.2E-08
ZNF239	-1.02	9.6E-08
ANKRD9	1.04	9.7E-08
FASTKD5	-1.00	1.0E-07
RAB3A	1.28	1.0E-07
MR1	-1.09	1.1E-07
PPP1R15A	0.96	1.1E-07
ZNF41	-1.09	1.2E-07
CARD6	-1.43	1.2E-07
TRIP11	-0.82	1.4E-07
MT-ND6	0.78	1.4E-07
HES1	0.99	1.4E-07
GFOD1	0.95	1.4E-07
UCP3	-2.38	1.4E-07
EGLN1	-0.86	1.4E-07
ZNF559	-1.22	1.5E-07
ZNF559	-1.22	1.5E-07
PSMG3-AS1	-1.64	1.5E-07
MEIS1	0.83	1.5E-07
NAB2	1.01	1.6E-07
FLVCR2	-2.16	1.6E-07
POLR1E	0.84	1.7E-07

PPAPDC1B	1.07	1.8E-07
SNHG1	0.80	1.8E-07
GNB3	-1.08	1.8E-07
HMCN1	-0.89	1.8E-07
CENPC	0.91	1.8E-07
SEMA3B	-1.52	1.8E-07
ZNF736	-0.89	1.8E-07
GAD1	1.21	1.9E-07
IGHMBP2	0.82	1.9E-07
ZSCAN5A	-1.40	2.0E-07
LYSMD1	0.81	2.0E-07
ZMYND8	-0.78	2.3E-07
MESDC2	-0.76	2.3E-07
SIRT5	-0.93	2.4E-07
TET1	0.80	2.4E-07
PLXNA4	-1.11	2.4E-07
SNX27	0.76	2.5E-07
ACTB	1.02	2.6E-07
ATF5	-1.06	2.6E-07
PLEKHH3	1.06	2.6E-07
MANSC1	-1.21	2.7E-07
FLRT2	-0.87	2.8E-07
LMBR1L	0.80	2.8E-07
AHNAK2	-0.85	2.8E-07
NSL1	-0.81	2.8E-07
CLK3	0.91	2.8E-07
TTC9C	0.88	2.9E-07
ZBTB12	-0.91	3.0E-07
FAM46C	0.99	3.3E-07
ZNF330	0.80	3.4E-07
HES7	1.77	3.6E-07
KLHL11	0.96	3.7E-07
TRNP1	-1.00	4.2E-07
AMMECR1	-0.77	4.2E-07
SAMD8	0.86	4.2E-07
NEFM	0.80	4.3E-07
DLX5	1.07	4.6E-07
CCDC162P	-1.23	4.7E-07
CCNB1IP1	0.79	4.8E-07
GADD45A	0.90	4.8E-07
IQCH-AS1	-1.21	5.0E-07
NUPL2	-0.82	5.3E-07
SF1	-0.75	5.4E-07
ZNF117	-1.15	5.5E-07
BRMS1L	-0.91	5.5E-07
MAN2B1	0.88	5.6E-07
SEC13	0.73	5.7E-07

SLC22A23	-0.87	5.9E-07
COL17A1	-0.99	6.1E-07
HECA	0.84	6.1E-07
FER	-0.86	6.2E-07
SPRY3	1.14	6.3E-07
TMEM209	-0.74	6.4E-07
HSD17B10	0.76	6.6E-07
SLC2A11	-1.18	6.6E-07
HOXD13	0.73	6.9E-07
KLHL42	-0.81	7.0E-07
LRP11	-0.75	7.1E-07
PDIA5	0.89	7.1E-07
C17orf97	-2.15	7.6E-07
BRCC3	-0.81	7.6E-07
ARRDC3	0.94	7.7E-07
POLR3D	0.73	8.0E-07
C2orf43	-1.00	8.2E-07
ADAM8	-1.90	8.2E-07
MPST	0.80	8.4E-07
MFSD5	-1.05	8.4E-07
ECSIT	0.85	8.5E-07
TMEM64	-0.93	8.6E-07
DDT	0.90	9.3E-07
FAM162A	-0.72	9.4E-07
LENG8-AS1	1.27	9.6E-07
LINC00115	1.50	9.9E-07
NSD1	-0.72	1.0E-06
HBP1	0.87	1.0E-06
RIPK4	0.96	1.0E-06
ANXA2R	1.83	1.0E-06
ANKRD34B	-1.11	1.0E-06
ZNF780B	-0.90	1.0E-06
DUSP6	1.34	1.0E-06
PPP1R15B	0.70	1.1E-06
PHKG1	1.86	1.1E-06
MEF2A	-0.73	1.1E-06
HSP90B1	0.72	1.2E-06
TADA2A	-0.76	1.2E-06
FBXL21	-0.98	1.2E-06
APPBP2	-0.74	1.2E-06
MPZ	1.38	1.3E-06
METTL21B	1.15	1.4E-06
RFX7	-0.84	1.4E-06
ZBTB3	1.35	1.4E-06
CCNO	1.02	1.4E-06
RAB7L1	0.79	1.4E-06
ICK	-0.79	1.4E-06

GATAD1	0.79	1.5E-06
IRGQ	0.76	1.5E-06
SOGA1	-0.84	1.5E-06
MT-ND1	-0.80	1.6E-06
ZMYND11	-0.73	1.6E-06
PPIL6	-1.41	1.7E-06
HMMR	-0.78	1.7E-06
LINC00174	-0.93	1.7E-06
FDFT1	0.68	1.7E-06
YAE1D1	-0.83	1.8E-06
ZNFX1	0.75	1.9E-06
HOXA11	0.88	1.9E-06
PTCHD4	-1.64	1.9E-06
ASH1L-AS1	1.65	2.0E-06
C1GALT1C1	-1.06	2.0E-06
C10orf12	0.77	2.0E-06
SUN2	0.77	2.0E-06
CHP1	0.70	2.1E-06
BCAS2	-0.72	2.1E-06
ZNF777	-0.76	2.1E-06
PSD4	-1.43	2.1E-06
VCPIP1	0.80	2.1E-06
ERN1	0.81	2.1E-06
UMPS	-0.79	2.3E-06
MTMR3	0.69	2.3E-06
NDUFA4L2	-2.13	2.3E-06
TBC1D3F	-1.01	2.3E-06
JRKL	0.82	2.3E-06
PPP1R26	0.76	2.3E-06
ANKEF1	-0.92	2.4E-06
ZDHHC11	-1.04	2.5E-06
CREBRF	0.87	2.5E-06
FEM1C	0.84	2.5E-06
RPAP2	-0.85	2.5E-06
OLIG2	1.48	2.5E-06
ZNF697	0.78	2.6E-06
RN7SL3	-2.08	2.7E-06
GTF3A	0.74	2.7E-06
LINC00858	-1.48	2.8E-06
RER1	-0.77	2.8E-06
SLC26A10	-0.90	2.8E-06
GATAD2B	0.69	2.8E-06
C9orf156	-1.14	2.9E-06
ZNF655	-0.79	2.9E-06
C17orf59	1.31	3.1E-06
PAPOLA	-0.66	3.1E-06
HECTD2	-0.88	3.2E-06

LINC01125	-1.27	3.2E-06
CD83	-0.91	3.2E-06
TEX261	-0.71	3.2E-06
ZNF644	-0.83	3.3E-06
F8	-1.20	3.3E-06
CPSF6	-0.66	3.4E-06
PROX1	0.99	3.4E-06
GAL	0.88	3.6E-06
NDUFA13	-1.41	3.6E-06
STK38	-0.73	3.7E-06
ZNF829	-0.73	3.7E-06
ZNF470	-0.99	3.7E-06
ANKRD27	0.69	3.7E-06
CHSY3	-1.12	3.8E-06
ST6GALNAC4	1.00	3.8E-06
USP40	-0.79	3.8E-06
ZNF292	-0.85	3.9E-06
ZEB1	0.90	4.0E-06
POU3F2	0.78	4.2E-06
TMEM159	1.19	4.2E-06
SCARA3	-0.84	4.3E-06
TOE1	-0.75	4.3E-06
TMEM80	-0.96	4.3E-06
GAS5	0.74	4.3E-06
ELOVL2	-0.70	4.5E-06
SNRPA	0.70	4.5E-06
MRPL55	0.72	4.6E-06
SLC25A25	0.86	4.7E-06
NDUFS8	0.83	4.8E-06
RBM26-AS1	-1.89	4.8E-06
NRN1	-0.87	4.8E-06
B4GALT6	-0.87	4.8E-06
RNF207	-0.98	4.9E-06
LINC00965	-1.61	4.9E-06
KBTBD6	0.75	4.9E-06
ZEB1-AS1	0.97	4.9E-06
DNAH6	-1.15	4.9E-06
GABARAPL1	0.75	4.9E-06
GRIN2C	-3.06	4.9E-06
GADD45B	0.90	4.9E-06
DNAH1	-0.95	5.2E-06
CABLES1	-0.79	5.3E-06
RBM4B	-0.97	5.5E-06
CAMLG	0.75	5.7E-06
C1R	-1.14	5.7E-06
MFAP3L	-0.81	5.9E-06
SAP30L	-0.87	6.2E-06

SYNE3	1.21	6.3E-06
ZNF418	-2.63	6.4E-06
POLR2J4	-1.62	6.4E-06
DNAJC19P5	2.46	6.5E-06
EPHA6	0.79	6.7E-06
C6orf211	-0.75	6.7E-06
FAM214B	0.94	6.7E-06
LINC00649	-0.85	6.8E-06
PLXDC1	-1.19	6.9E-06
ZFP36	0.87	6.9E-06
RABAC1	0.71	7.0E-06
TMEM127	-0.76	7.1E-06
ZNF66	-1.82	7.1E-06
KLF4	0.95	7.1E-06
MVB12A	0.82	7.1E-06
ITGA8	-0.78	7.2E-06
EPDR1	-0.87	7.3E-06
TEF	0.83	7.3E-06
EIF3C	0.79	7.8E-06
VAX1	-1.85	7.9E-06
ZNF542	-1.31	7.9E-06
PNP	0.84	8.0E-06
HMBOX1	0.74	8.0E-06
ZFAND2B	0.97	8.2E-06
PHF20L1	-0.72	8.2E-06
ZUFSP	0.78	8.2E-06
FAM66C	-1.37	8.3E-06
CCP110	0.72	8.3E-06
TICRR	0.68	8.4E-06
PAIP2B	0.69	8.4E-06
PRRT4	-0.81	8.4E-06
PPM1J	1.21	8.5E-06
APCDD1	-0.93	8.5E-06
TUBE1	0.78	8.7E-06
CLDND2	1.57	8.8E-06
HTRA1	-0.72	9.0E-06
WHAMM	0.75	9.0E-06
BRICD5	-1.47	9.0E-06
SMYD5	0.73	9.1E-06
STON2	0.76	9.1E-06
FLT1	-0.79	9.2E-06
MLH3	-0.72	9.2E-06
RAB33B	0.86	9.3E-06
DACT3	1.11	9.4E-06
C6orf136	0.81	1.0E-05
TIGD1	0.84	1.0E-05
ZBTB38	-0.87	1.0E-05

ALG6	-0.83	1.0E-05
NDUFS6	0.68	1.0E-05
OGT	-0.66	1.0E-05
LCA5L	-2.68	1.0E-05
DCBLD2	-0.69	1.0E-05
CHRNA10	-3.00	1.0E-05
ZFHX2	-1.09	1.1E-05
GAN	0.70	1.1E-05
DNAH10OS	0.98	1.2E-05
CLN5	-1.02	1.2E-05
JOSD1	-0.72	1.2E-05
C22orf39	0.83	1.2E-05
LXN	-0.84	1.2E-05
FKBP14	0.79	1.2E-05
HSD17B7P2	1.50	1.2E-05
FBXO4	-1.45	1.3E-05
ANTXR1	-0.66	1.3E-05
YPEL3	1.01	1.3E-05
NTMT1	0.77	1.3E-05
WASH7P	0.96	1.3E-05
SLC25A5	0.66	1.3E-05
ZDHHC24	0.93	1.3E-05
RGS16	0.96	1.3E-05
RPLP2	0.69	1.3E-05
RAB23	-0.74	1.3E-05
KLHL24	0.85	1.3E-05
ABI3BP	-1.43	1.3E-05
LINC00641	-0.68	1.4E-05
IQCC	0.86	1.4E-05
TMEM198B	-0.70	1.4E-05
PYGO1	-0.89	1.4E-05
MTERF	-0.96	1.4E-05
SLC38A10	-0.72	1.4E-05
MSTO2P	0.98	1.4E-05
SLC29A3	-1.11	1.4E-05
PMEPA1	-0.99	1.5E-05
FASN	0.69	1.5E-05
BEND3	-0.80	1.6E-05
H2AFY2	-0.65	1.6E-05
LYPD6	-0.94	1.6E-05
TTC12	-0.79	1.6E-05
SNHG3	0.90	1.6E-05
CCDC138	-0.73	1.6E-05
NETO2	-0.65	1.6E-05
HCG18	-0.71	1.7E-05
PTK2B	-0.93	1.7E-05
MYO15B	-0.95	1.7E-05

SDF2L1	1.35	1.7E-05
MUM1L1	-0.95	1.7E-05
CD99	0.71	1.7E-05
RAG1	-2.33	1.7E-05
MYOM2	-1.17	1.7E-05
KPNA6	0.64	1.7E-05
TIPARP	0.74	1.8E-05
MRPL52	0.75	1.8E-05
NDUFB8	1.41	1.8E-05
HIST1H4E	-2.47	1.8E-05
HDX	-1.28	1.8E-05
SSH1	-0.64	1.8E-05
BOLA3-AS1	0.72	1.9E-05
SOX9	0.88	1.9E-05
IER3	0.85	1.9E-05
CCER2	-2.00	1.9E-05
ALDH1L2	-0.63	1.9E-05
ADAM21	-2.37	2.0E-05
KATNAL2	-1.10	2.0E-05
ENO3	-0.88	2.0E-05
C7orf49	-0.72	2.0E-05
TSPYL1	0.68	2.0E-05
RDM1	0.88	2.0E-05
PDK1	-0.74	2.0E-05
THNSL1	-0.84	2.1E-05
TMSB4X	0.69	2.1E-05
CYP26A1	1.14	2.1E-05
ACLY	0.65	2.1E-05
EDARADD	-0.95	2.1E-05
EEF1A2	0.90	2.2E-05
PDP1	0.74	2.2E-05
RPS15	0.70	2.2E-05
RPUSD3	0.69	2.3E-05
ZIK1	-1.20	2.3E-05
ARHGAP31	-0.82	2.3E-05
RPLP1	1.01	2.3E-05
LHX9	-0.99	2.3E-05
SAMD11	-0.70	2.3E-05
MANF	0.70	2.3E-05
HOXC13	0.78	2.3E-05
COX18	0.83	2.4E-05
MOCOS	-1.24	2.4E-05
SPDL1	-0.67	2.4E-05
YOD1	0.75	2.4E-05
CSRP2BP	-0.78	2.4E-05
CNKSR3	0.65	2.4E-05
LRP8	0.67	2.4E-05

ASF1A	-0.74	2.5E-05
FZD8	0.81	2.5E-05
CYB5D2	-0.92	2.5E-05
ORAOV1	-0.70	2.5E-05
HOXA-AS2	0.86	2.5E-05
DUSP8P5	1.11	2.5E-05
ZNF75D	-0.70	2.5E-05
PLAGL2	-0.63	2.5E-05
CXorf38	0.84	2.6E-05
AGGF1	-0.65	2.6E-05
PPP6R3	-0.61	2.6E-05
DOCK8	-0.91	2.6E-05
YPEL2	0.71	2.6E-05
OGFR	0.72	2.6E-05
SRBD1	-0.78	2.7E-05
TAF1A	0.91	2.7E-05
RBM47	-0.97	2.8E-05
HLCS	-0.78	2.8E-05
APH1B	-0.80	2.8E-05
ASF1B	-0.67	2.8E-05
CSF1	-0.78	3.0E-05
GPC4	0.63	3.0E-05
NFKB1	-0.82	3.0E-05
HYLS1	-0.93	3.0E-05
ZNF554	-0.81	3.0E-05
TPPP	0.80	3.1E-05
SHISA4	-1.96	3.1E-05
SLTM	-0.61	3.1E-05
GTF2H2	0.85	3.1E-05
FNTB	-1.12	3.2E-05
PTMA	0.62	3.2E-05
PTRH2	-0.65	3.2E-05
NMRK1	-0.99	3.2E-05
IL18R1	-1.00	3.3E-05
AIFM2	-2.10	3.3E-05
GOLGA8A	-0.62	3.3E-05
BABAM1	1.07	3.3E-05
CROCCP2	0.72	3.4E-05
TRIM52-AS1	0.75	3.5E-05
SNHG10	0.82	3.5E-05
WDR33	-0.60	3.7E-05
HUNK	-0.78	3.7E-05
ARL16	0.74	3.8E-05
ULBP2	0.87	3.8E-05
ZSCAN25	-0.73	3.8E-05
LONRF2	-0.66	3.9E-05
ZNF112	-1.05	3.9E-05

SYNM	-0.66	3.9E-05
SLC31A1	0.64	3.9E-05
ARHGDIA	0.79	4.1E-05
NDE1	0.63	4.1E-05
USP31	0.71	4.1E-05
RRM2	0.60	4.1E-05
SNRPB	0.94	4.1E-05
ZNF852	-1.30	4.1E-05
SSH3	-1.89	4.2E-05
ZNF780A	-0.82	4.3E-05
HOXA9	0.75	4.3E-05
ZNF549	-0.83	4.3E-05
HLA-E	0.61	4.4E-05
ZNF140	-0.85	4.6E-05
PAFAH2	-0.88	4.6E-05
ZDBF2	-0.77	4.7E-05
SLC30A6	-0.72	4.7E-05
SMAD7	0.72	4.8E-05
SLC9A2	-1.64	4.8E-05
ZNF274	-0.62	4.9E-05
PPIC	0.78	5.0E-05
GOLGA2	0.66	5.0E-05
GPRASP1	-1.60	5.1E-05
RSRC2	0.78	5.1E-05
AKAP17A	0.67	5.1E-05
C8orf31	-1.31	5.1E-05
RPGRIP1L	-0.71	5.1E-05
GBAP1	-0.95	5.2E-05
GTPBP1	0.70	5.3E-05
SMIM19	-0.81	5.4E-05
PIK3CA	4.25	5.4E-05
TTC30A	-0.90	5.4E-05
FAXC	-0.68	5.5E-05
SMARCAD1	-0.61	5.6E-05
WBSCR22	0.64	5.6E-05
ERVK3-1	-0.65	5.7E-05
ELK4	0.84	5.8E-05
RNF6	-0.61	5.8E-05
KMT2E-AS1	1.35	5.9E-05
LHFPL4	-0.86	6.0E-05
EML2	0.62	6.0E-05
ZNF804A	-1.66	6.0E-05
ASNSD1	-0.65	6.1E-05
PANK1	-0.76	6.1E-05
DZIP3	-0.66	6.1E-05
ALKBH6	0.76	6.1E-05
SP8	1.19	6.4E-05

DDIT4	0.67	6.4E-05
F3	1.07	6.4E-05
LHX4	0.82	6.5E-05
BRCA1	-0.66	6.5E-05
DPH7	0.68	6.5E-05
ADAT2	0.67	6.5E-05
ZNF577	-0.87	6.6E-05
NUDT22	0.92	6.6E-05
E2F2	0.70	6.7E-05
ZNF480	-0.76	6.8E-05
AP3B2	-0.92	6.9E-05
C17orf80	-0.74	7.0E-05
LINC00202-1	-0.80	7.2E-05
ERLIN1	0.60	7.2E-05
HN1	0.63	7.3E-05
ANKRD11	-0.59	7.4E-05
FAM114A2	-0.66	7.5E-05
JAGN1	-0.80	7.5E-05
SHISA2	0.78	7.5E-05
NPM3	0.61	7.5E-05
HABP4	0.78	7.5E-05
SERTAD2	0.73	7.5E-05
RRP15	-0.64	7.6E-05
DTX3L	-1.21	7.6E-05
CLDN15	-0.73	7.7E-05
ZNF649	-0.76	7.8E-05
FAM124A	-1.52	7.9E-05
IL11RA	-0.80	7.9E-05
HNRNPU-AS1	0.64	7.9E-05
LINC00632	0.93	7.9E-05
ALPK2	-1.78	7.9E-05
EID2B	0.97	8.0E-05
HCG11	-0.80	8.1E-05
ZWINT	-0.59	8.1E-05
ZBTB16	1.03	8.1E-05
SLC1A1	-0.75	8.2E-05
AAR2	-0.66	8.2E-05
LRRC8D	-0.78	8.4E-05
VARS	0.61	8.6E-05
RBM11	0.75	8.6E-05
TMEM62	-1.01	8.8E-05
LAMP1	0.63	8.8E-05
USP49	0.64	8.8E-05
NHP2	0.62	8.8E-05
TRMT11	0.65	8.8E-05
DDX6	-0.62	8.8E-05
TTC18	-1.08	8.8E-05

NRIP1	-0.63	8.9E-05
CCDC61	0.98	8.9E-05
CCDC81	2.28	9.0E-05
CD101	-1.14	9.0E-05
KDELR1	0.58	9.0E-05
UBAP1L	-1.02	9.0E-05
MBLAC2	-0.72	9.0E-05
ANKMY1	-0.80	9.1E-05
ZNF85	-0.75	9.1E-05
ANGPT1	-0.97	9.2E-05
TBC1D22A	-0.68	9.2E-05
LINC00638	-1.76	9.3E-05
SIGMAR1	0.59	9.4E-05
PSMG4	0.77	9.5E-05
ANTXR2	-0.67	9.5E-05
CIRBP-AS1	-1.14	9.7E-05
GTF2H1	-0.65	9.7E-05
ISCA1	-0.69	9.8E-05
XYLB	-0.71	9.9E-05
DPP7	-1.07	1.0E-04
C10orf118	0.73	1.0E-04
SLC35A5	-0.76	1.0E-04
ITGA2	-0.69	1.0E-04
DHFRL1	-1.04	1.0E-04
CCRN4L	0.86	1.0E-04
DENND6B	-1.13	1.0E-04
SCGB2B2	-1.39	1.0E-04
NEU3	0.64	1.0E-04
EPC1	-0.63	1.1E-04
RAPGEF5	0.64	1.1E-04
GUCY1A3	-0.76	1.1E-04
BASP1	0.79	1.1E-04
KIAA1211L	-1.63	1.1E-04
SNHG15	0.59	1.1E-04
TRPC1	-0.74	1.1E-04
AEN	-0.61	1.1E-04
CXorf40A	-0.73	1.1E-04
ATAD5	0.59	1.1E-04
AP5B1	-0.82	1.1E-04
PALMD	-0.95	1.1E-04
HCFC2	0.69	1.1E-04
SYNC	-1.76	1.1E-04
NUAK2	1.17	1.1E-04
PHC3	0.64	1.1E-04
IER5	0.74	1.1E-04
RNF128	-0.71	1.1E-04
KDM7A	0.67	1.2E-04

TMPO-AS1	-0.81	1.2E-04
BARHL2	1.38	1.2E-04
TRMT10A	-0.81	1.2E-04
OLFML2A	-1.08	1.2E-04
KLF6	0.65	1.2E-04
PHACTR1	0.70	1.2E-04
PUSL1	0.84	1.2E-04
ST6GALNAC3	-0.79	1.2E-04
KIAA1875	-1.58	1.3E-04
NYNRIN	-0.70	1.3E-04
HMGB3	0.58	1.3E-04
SMOC2	-2.11	1.3E-04
CCDC9	0.73	1.3E-04
BTRC	-0.63	1.3E-04
LIAS	0.91	1.3E-04
BEND5	-1.62	1.3E-04
HIVEP3	-0.72	1.4E-04
RPL18	0.64	1.4E-04
CTU2	0.83	1.4E-04
ZNF229	-0.83	1.4E-04
AARSD1	1.20	1.4E-04
CDK17	0.65	1.4E-04
COL7A1	-0.82	1.4E-04
PPP1R26-AS1	-1.24	1.4E-04
RIMKLA	-0.98	1.4E-04
ZNF583	-0.87	1.4E-04
LINC00839	-0.94	1.5E-04
COTL1	0.72	1.5E-04
AK9	-1.05	1.5E-04
ZNRD1	0.78	1.5E-04
ZNF484	0.99	1.5E-04
NUP214	-0.58	1.5E-04
NAT6	0.85	1.5E-04
HEY2	1.06	1.5E-04
ZNF217	0.74	1.5E-04
ZNF793	-0.69	1.5E-04
AMER1	-0.67	1.5E-04
LPCAT4	0.74	1.5E-04
TFRC	-0.61	1.5E-04
SNCAIP	-0.85	1.5E-04
LYPLAL1	-0.71	1.5E-04
CDKN1B	0.58	1.5E-04
RAD52	0.63	1.5E-04
COL9A2	-0.79	1.5E-04
FOXC2	1.18	1.5E-04
SLC25A24	-0.65	1.6E-04
BCL9L	0.74	1.6E-04

HS3ST3A1	0.78	1.6E-04
PPHLN1	-0.59	1.6E-04
IBA57	-0.69	1.6E-04
KCNB1	-1.32	1.7E-04
SUSD2	-2.18	1.7E-04
DPY19L2	-1.05	1.7E-04
MIR34A	-1.60	1.7E-04
EP400NL	-0.66	1.7E-04
RIC8B	-0.68	1.7E-04
MTMR1	-0.59	1.7E-04
N6AMT2	-1.07	1.7E-04
ZNF688	-1.13	1.7E-04
C5	-1.89	1.8E-04
GJA5	-1.40	1.8E-04
C5orf28	-0.95	1.8E-04
KIAA1407	-0.97	1.8E-04
SLC38A9	-0.66	1.8E-04
PRPSAP1	0.59	1.8E-04
IFIT5	-0.61	1.9E-04
HSPA2	-0.72	1.9E-04
LIG4	-0.69	1.9E-04
GPR27	-0.70	1.9E-04
ASIC3	-0.97	1.9E-04
VAPB	-0.65	1.9E-04
PAWR	0.63	1.9E-04
PARD6B	0.97	2.0E-04
UFSP1	-1.76	2.0E-04
ZNF708	-0.81	2.0E-04
CEACAM19	-0.73	2.0E-04
DUSP7	0.69	2.0E-04
CHCHD2	0.90	2.0E-04
LYRM7	-0.69	2.0E-04
PIGC	-0.68	2.0E-04
SENP5	0.63	2.1E-04
PRX	0.79	2.1E-04
AGER	-1.04	2.1E-04
FHL3	0.93	2.1E-04
FBXO46	0.75	2.1E-04
ANK3	-0.59	2.1E-04
SEMA7A	0.84	2.1E-04
DTYMK	0.71	2.1E-04
MXD1	0.68	2.2E-04
KIAA0232	-0.64	2.2E-04
TMEM254-AS1	-1.34	2.2E-04
KIAA1549L	-0.63	2.2E-04
RICTOR	0.69	2.2E-04
ZC3H12A	0.74	2.2E-04

ZNF790	-0.74	2.2E-04
TLR3	-1.37	2.3E-04
SIK2	-0.63	2.3E-04
N4BP2	-0.66	2.3E-04
CDC27	-0.59	2.3E-04
ADAMTS10	-0.70	2.3E-04
ATP1A3	0.68	2.3E-04
BCHE	-0.74	2.3E-04
CHURC1	-0.96	2.3E-04
ZNF121	0.59	2.3E-04
ZCWPW1	-1.00	2.3E-04
ID4	0.66	2.4E-04
KBTBD11	-0.83	2.4E-04
UBXN8	-0.70	2.4E-04
TUBB2B	0.61	2.4E-04
C9orf37	0.79	2.4E-04
DDAH2	0.74	2.5E-04
SIRPA	-0.65	2.5E-04
SGK3	0.98	2.5E-04
GCDH	0.66	2.6E-04
INPP5D	-0.73	2.6E-04
IGSF8	0.73	2.6E-04
DNM1P35	-1.82	2.6E-04
NRP2	0.63	2.6E-04
SLC26A7	-0.94	2.6E-04
GTPBP3	0.64	2.6E-04
CENPF	-0.58	2.6E-04
TMEM194B	-0.99	2.6E-04
IGDCC4	-0.69	2.6E-04
HIST3H2A	1.22	2.6E-04
SNED1	-0.86	2.7E-04
TDRD6	-1.38	2.7E-04
BNIP1	0.70	2.7E-04
MROH6	-1.08	2.7E-04
ITPKC	0.66	2.7E-04
CCDC85B	1.55	2.7E-04
LIPT2	-1.01	2.7E-04
ALDH1A3	-0.70	2.7E-04
AKAP8L	0.59	2.7E-04
NDUFS7	0.66	2.7E-04
MAF	0.64	2.7E-04
ZNF765	-0.78	2.8E-04
ZNF827	-0.78	2.8E-04
HSPA1B	0.65	2.8E-04
SLC19A2	0.69	2.8E-04
TTN-AS1	-0.73	2.8E-04
PGBD2	-1.33	2.9E-04

STARD5	0.84	2.9E-04
RHOQP1	1.40	2.9E-04
VSTM4	-0.78	2.9E-04
CSPG5	-0.76	2.9E-04
FAM69A	-0.64	3.0E-04
DACT1	0.68	3.0E-04
UBE2S	0.75	3.0E-04
CISH	0.95	3.0E-04
RPRD2	-0.60	3.0E-04
CRELD1	0.64	3.0E-04
ZNF354C	-0.93	3.0E-04
TMEM165	-0.62	3.0E-04
C12orf65	0.66	3.0E-04
PIM3	0.66	3.1E-04
TRPT1	0.84	3.1E-04
DPYSL4	-1.01	3.1E-04
BUD13	0.60	3.1E-04
C2CD2	-0.63	3.1E-04
NAALADL1	1.78	3.2E-04
TIRAP	-0.77	3.2E-04
STRADA	0.79	3.2E-04
STAG1	-0.63	3.2E-04
CFI	-0.99	3.2E-04
KIF24	-0.70	3.2E-04
ARHGAP28	-0.73	3.3E-04
GPRIN3	0.60	3.3E-04
PHF3	0.61	3.3E-04
THRB	-0.61	3.3E-04
TMEM18	-0.61	3.3E-04
MSRB3	-0.61	3.3E-04
DRAM1	-0.99	3.3E-04
SCAMP5	-0.65	3.3E-04
ZNF124	-1.33	3.4E-04
SLC37A2	1.06	3.4E-04
MED17	-0.66	3.4E-04
RASA4CP	-1.98	3.5E-04
USB1	0.62	3.6E-04
MBNL1-AS1	0.91	3.6E-04
TRPA1	-1.22	3.6E-04
PRICKLE4	-0.81	3.6E-04
SNAPC2	1.01	3.6E-04
PIH1D2	-1.83	3.7E-04
PCGF1	0.62	3.7E-04
SNX18P7	-1.72	3.7E-04
PHLDB3	0.76	3.7E-04
WDR63	-0.81	3.8E-04
STOX1	-0.97	3.8E-04

POLR3B	-0.62	3.9E-04
TMEM79	0.80	3.9E-04
GPER1	-1.43	3.9E-04
TROVE2	0.59	3.9E-04
ADSSL1	1.00	3.9E-04
GLRX5	0.59	4.0E-04
FZD5	0.62	4.0E-04
MEF2C	-0.61	4.0E-04
NKX2-8	1.49	4.0E-04
TBC1D8	0.60	4.1E-04
DYNLL1-AS1	-1.03	4.1E-04
SNORA75	1.69	4.1E-04
CD55	0.61	4.2E-04
EMP1	0.86	4.2E-04
CMBL	0.58	4.2E-04
RGMB	0.64	4.3E-04
ZNF615	0.66	4.4E-04
CCNJL	-1.19	4.4E-04
DISP1	-0.68	4.4E-04
SLC25A1	0.62	4.5E-04
MYH15	-1.36	4.5E-04
ETNK1	-0.62	4.5E-04
GDNF-AS1	-0.85	4.5E-04
ZNF581	0.90	4.5E-04
PEX3	-0.60	4.6E-04
SRGAP2C	0.83	4.6E-04
CCDC89	-1.64	4.6E-04
PPDPF	0.87	4.6E-04
C2orf72	-0.74	4.6E-04
CREG2	1.42	4.7E-04
ANKRD31	-1.62	4.7E-04
ITPRIP	0.65	4.7E-04
NECAP2	-0.61	4.7E-04
C2CD2L	0.78	4.8E-04
ZNF202	-0.65	4.9E-04
RGPD5	1.65	4.9E-04
DLX2	0.63	5.0E-04
ATL1	-0.93	5.0E-04
COLGALT1	0.61	5.0E-04
NKX2-4	-0.77	5.0E-04
RND1	1.00	5.0E-04
TXLNB	-0.95	5.1E-04
MAFK	0.90	5.1E-04
FBLN2	1.77	5.2E-04
NPC1L1	-1.20	5.2E-04
FAM86HP	-1.73	5.2E-04
RUVBL2	0.62	5.3E-04

RLTPR	0.95	5.3E-04
ZNF519	-0.70	5.3E-04
C22orf26	-1.63	5.4E-04
AKIP1	0.67	5.5E-04
ZFP36L1	0.76	5.5E-04
ZNF782	-1.01	5.6E-04
LYPD3	1.13	5.7E-04
ADM	0.65	5.7E-04
FGFR3	0.59	5.8E-04
ZNF32	-0.70	5.8E-04
SIX4	0.61	5.8E-04
DLX6	0.60	5.9E-04
AMH	-1.02	5.9E-04
TEX264	-0.61	5.9E-04
EIF4A1	0.62	5.9E-04
FOS	0.92	5.9E-04
HIST1H1E	-2.09	5.9E-04
CDK5R1	0.69	5.9E-04
DDTL	-0.97	5.9E-04
CBX7	-0.77	5.9E-04
PIGP	-0.62	6.0E-04
ARNT2	-0.60	6.0E-04
C19orf82	-1.39	6.1E-04
TOR1A	-0.63	6.2E-04
CCDC110	-1.32	6.2E-04
DHRS7B	-0.71	6.2E-04
CCDC102A	-1.10	6.3E-04
URGCP	-0.60	6.3E-04
USP4	0.58	6.4E-04
UBOX5	0.65	6.5E-04
GUCY1B2	-1.16	6.6E-04
PKD1P6	0.69	6.6E-04
C21orf67	-1.31	6.6E-04
RAD51C	-0.68	6.7E-04
C3orf62	-1.15	6.7E-04
KLHL3	-0.62	6.7E-04
IRAK1BP1	-0.62	6.7E-04
GCNT1	0.70	6.8E-04
OR2A1-AS1	-0.98	6.8E-04
ZNF574	0.70	6.8E-04
WASH6P	0.80	6.8E-04
CASP8	-0.59	6.9E-04
CCDC19	1.41	7.0E-04
TMEM254	-0.67	7.0E-04
HOTTIP	-1.05	7.0E-04
HOXA7	-0.71	7.2E-04
ZNF394	0.74	7.3E-04

ZNF404	-0.94	7.3E-04
DGKZ	0.65	7.3E-04
AKIRIN2	0.65	7.3E-04
ARID3A	0.64	7.4E-04
C16orf52	-0.74	7.4E-04
TMEM5	-0.69	7.5E-04
CNEP1R1	0.77	7.5E-04
CTBP1-AS2	-0.59	7.5E-04
TP53RK	-0.73	7.6E-04
MRPL43	0.59	7.6E-04
STAC3	-1.02	7.7E-04
HOMEZ	-0.60	7.8E-04
BCDIN3D	-0.86	7.9E-04
RLIM	0.59	7.9E-04
CTSO	-0.91	8.0E-04
GPX1	0.67	8.0E-04
TMEM132B	-0.84	8.1E-04
CCDC47	-0.63	8.1E-04
TNS3	-0.60	8.1E-04
PDRG1	0.72	8.1E-04
ENPP5	0.73	8.1E-04
LRRC66	-1.85	8.1E-04
IRS2	0.63	8.2E-04
C15orf57	0.67	8.2E-04
ZNF699	0.86	8.2E-04
CARF	-0.84	8.3E-04
DHRSX	-0.82	8.3E-04
MALAT1	0.71	8.4E-04
TRAM2-AS1	-0.88	8.5E-04
SLC16A14	-0.64	8.5E-04
MESP2	1.72	8.6E-04
TK2	-0.86	8.7E-04
CCDC14	-0.59	8.9E-04
RBM3	0.64	8.9E-04
EEF1A1P19	1.74	9.0E-04
TMEM39B	-0.71	9.2E-04
ARG2	0.62	9.2E-04
ZNF107	-0.64	9.2E-04
PTER	-0.63	9.2E-04
LINC01002	0.90	9.2E-04
PRIMA1	-0.78	9.3E-04
RMND1	0.61	9.3E-04
TENC1	-0.65	9.4E-04
KDM4D	-0.87	9.5E-04
BCL7B	-0.73	9.5E-04
POLN	-0.76	9.8E-04
ZWILCH	-0.62	9.8E-04

PAX1	-0.88	9.8E-04
NKX6-1	0.79	9.9E-04
XRCC4	-0.79	9.9E-04
CACNG8	-0.93	1.0E-03
PTPRZ1	-0.77	1.0E-03
SLC6A8	0.61	1.0E-03
TMEM98	-0.60	1.0E-03
TLE3	0.58	1.0E-03
PGM2L1	0.64	1.0E-03
ZNF681	-1.23	1.0E-03
RIPK2	0.60	1.0E-03
THSD7A	-1.06	1.0E-03
EIF4A2	0.60	1.0E-03
RMRP	-3.25	1.0E-03
ZNF724P	0.75	1.0E-03
IFIT1	-0.71	1.0E-03
NUPR1	-1.05	1.1E-03
FBXO27	0.76	1.1E-03
SSPO	-0.88	1.1E-03
NACC2	-0.62	1.1E-03
LRRIQ1	-1.10	1.1E-03
FAM193B	0.71	1.1E-03
ERV3-1	-0.74	1.1E-03
FTX	-1.69	1.1E-03
HCN2	0.66	1.1E-03
NXPH3	0.92	1.1E-03
C14orf28	1.01	1.1E-03
THAP3	0.70	1.1E-03
WDR31	-1.07	1.1E-03
FANCM	0.61	1.1E-03
FBXL2	0.71	1.1E-03
SNORD117	1.07	1.1E-03
SLC16A6	-1.12	1.1E-03
PHF1	0.62	1.1E-03
DEPTOR	0.63	1.1E-03
ZNF570	0.60	1.1E-03
BAMBI	0.63	1.2E-03
TNFRSF12A	1.16	1.2E-03
HERC6	-0.68	1.2E-03
OXTR	0.87	1.2E-03
DLL1	0.62	1.2E-03
CDC37L1	0.67	1.2E-03
ZNF296	0.87	1.2E-03
SCN5A	-0.94	1.2E-03
ZNF783	-0.65	1.2E-03
TTPAL	-0.72	1.2E-03
FAM118B	-0.75	1.2E-03

ANKDD1A	1.30	1.2E-03
VPS18	-0.72	1.2E-03
ING3	-0.65	1.3E-03
ELOF1	0.62	1.3E-03
ZNF853	-0.61	1.3E-03
TTLL11	-0.79	1.3E-03
VCPKMT	0.77	1.3E-03
DUSP10	0.68	1.3E-03
C15orf61	0.83	1.3E-03
SDCBP2	1.13	1.3E-03
MORN1	-0.76	1.3E-03
PYROXD1	-0.73	1.3E-03
SLC9A8	-0.63	1.3E-03
KIAA1045	-1.27	1.3E-03
ATG4D	0.66	1.3E-03
RBAK	-0.62	1.3E-03
PHF21B	0.76	1.3E-03
SRP14-AS1	-0.91	1.3E-03
ZNF471	-0.74	1.3E-03
MAP2K7	0.62	1.4E-03
TLR5	-1.59	1.4E-03
TMEM67	-0.60	1.4E-03
NAT9	0.61	1.4E-03
MAD2L2	0.61	1.4E-03
ZNF627	0.76	1.4E-03
LINC00176	-1.32	1.4E-03
ITPRIPL2	-0.61	1.4E-03
PEX11A	-0.66	1.4E-03
TRAM1	-0.64	1.4E-03
ISL2	0.64	1.4E-03
DGCR11	1.25	1.4E-03
CALHM2	0.61	1.4E-03
PDE4B	-0.87	1.5E-03
SBNO2	0.70	1.5E-03
C12orf5	-0.66	1.5E-03
NAT1	-0.89	1.5E-03
CALCB	0.72	1.5E-03
AP4S1	-1.44	1.5E-03
RASSF4	-0.75	1.5E-03
TXNDC5	0.97	1.5E-03
MIEF2	0.64	1.5E-03
SPPL2A	-0.65	1.5E-03
ZNF268	-0.62	1.5E-03
TBX2	0.66	1.5E-03
MAMDC4	-0.90	1.5E-03
PPARGC1A	0.76	1.5E-03
TBC1D17	0.68	1.6E-03

NEK11	-1.31	1.6E-03
LINC-PINT	1.10	1.6E-03
HOXC8	-0.59	1.6E-03
NEXN	-0.59	1.6E-03
CLGN	-0.83	1.6E-03
TNFAIP8L3	1.55	1.6E-03
ARMCX4	-1.37	1.6E-03
RAB36	-0.76	1.6E-03
CEP97	0.59	1.6E-03
RGS7BP	-0.92	1.6E-03
DHDH	1.13	1.6E-03
LINC01023	1.66	1.6E-03
ACSS3	0.73	1.7E-03
DOLK	-0.71	1.7E-03
XKR5	-1.14	1.7E-03
PDCD7	0.59	1.7E-03
MAFF	1.45	1.7E-03
f	0.62	1.7E-03
EDA2R	-0.70	1.7E-03
GEM	0.71	1.7E-03
CUZD1	-1.12	1.7E-03
SCN2A	-1.59	1.7E-03
WNK3	0.62	1.7E-03
SDAD1P1	-1.15	1.7E-03
MAPKAPK5-AS1	0.78	1.7E-03
SHROOM1	-0.69	1.7E-03
ACTRT3	-1.53	1.8E-03
METTL25	-1.01	1.8E-03
KLF16	0.69	1.8E-03
HIST1H2BJ	1.06	1.8E-03
PTHLH	1.15	1.8E-03
TAGLN	-1.09	1.8E-03
SPSB2	0.86	1.8E-03
LIMK1	0.71	1.8E-03
COX5A	0.72	1.8E-03
RPS6KB2	0.60	1.8E-03
PAXIP1-AS2	-0.95	1.8E-03
MURC	-1.03	1.9E-03
GDNF	-0.85	1.9E-03
AOC2	0.64	1.9E-03
CYP4X1	-0.62	1.9E-03
TPH1	-1.71	1.9E-03
IQCH	-1.00	1.9E-03
SHC2	-0.79	1.9E-03
DHRS1	0.68	1.9E-03
PTPMT1	-0.80	2.0E-03
FIGN	0.63	2.0E-03

S1PR2	0.84	2.0E-03
KRAS	1.02	2.0E-03
ANKHD1-EIF4EBP3	-1.63	2.1E-03
TDRD12	-1.17	2.1E-03
SKIL	0.67	2.1E-03
TMEM134	0.74	2.1E-03
MGMT	0.65	2.1E-03
FAM86B3P	0.58	2.1E-03
TRIM36	0.62	2.1E-03
ZNF695	1.16	2.1E-03
ALG11	-0.86	2.1E-03
UBALD2	0.83	2.1E-03
P2RY1	0.67	2.1E-03
HSF4	-0.93	2.1E-03
STK17B	0.69	2.1E-03
MYLIP	0.67	2.2E-03
ZNF528	-0.80	2.2E-03
TEAD4	0.61	2.2E-03
PEX6	-0.58	2.2E-03
CHCHD5	0.76	2.2E-03
MAEA	-0.66	2.2E-03
KCNMB3	-1.35	2.2E-03
ZNF425	-1.28	2.2E-03
SLC7A11	-0.70	2.2E-03
TMEM117	-0.62	2.2E-03
PKIA	-0.60	2.2E-03
GNG5	0.63	2.2E-03
MSTN	-1.33	2.2E-03
TRAF1	-0.99	2.2E-03
GRB14	0.72	2.2E-03
HELQ	-0.72	2.2E-03
SCAND2P	0.62	2.2E-03
PRSS27	-1.12	2.3E-03
MDH1B	-1.75	2.3E-03
RHBDF1	0.60	2.3E-03
TNFAIP8L1	0.71	2.3E-03
C9orf89	0.85	2.3E-03
DCHS1	-0.94	2.3E-03
CDKN2D	0.83	2.3E-03
TMEM168	-0.65	2.3E-03
OPHN1	0.58	2.3E-03
ARFRP1	0.66	2.3E-03
PPP4R1L	0.67	2.3E-03
WDR4	0.58	2.3E-03
SLC25A19	0.63	2.3E-03
TUSC1	-0.71	2.3E-03

VWDE	0.61	2.3E-03
BEAN1	-1.21	2.3E-03
NFIL3	0.64	2.4E-03
AMY2B	-1.12	2.4E-03
LPAR3	0.61	2.4E-03
CASC2	-0.99	2.5E-03
NFKBIB	0.74	2.5E-03
SHPK	1.21	2.5E-03
FBXL19-AS1	-0.74	2.5E-03
SAMD1	0.61	2.6E-03
ZNF836	-1.44	2.6E-03
ZNF502	-0.96	2.6E-03
TPST1	-0.59	2.6E-03
IL6R	0.60	2.6E-03
MRPL1	-0.77	2.6E-03
SCN1B	0.92	2.6E-03
SDCBP2-AS1	-1.31	2.6E-03
NFATC4	-0.61	2.6E-03
PAM16	1.05	2.6E-03
ZNF862	-0.61	2.6E-03
FZD10-AS1	-1.46	2.6E-03
NHLH2	-1.82	2.7E-03
RHBDL3	-0.59	2.7E-03
C1RL	-0.83	2.7E-03
PLEKHG4B	-0.65	2.7E-03
XRCC6BP1	0.69	2.8E-03
THEM4	-0.60	2.8E-03
DTX4	-0.61	2.8E-03
SH2B3	0.58	2.8E-03
LDOC1	0.62	2.8E-03
ITGB1BP2	-1.17	2.8E-03
LRRC17	-1.19	2.9E-03
AP4E1	-0.66	2.9E-03
TEAD3	0.61	2.9E-03
SREBF1	-0.61	2.9E-03
DLL4	1.03	2.9E-03
NHSL2	-0.84	2.9E-03
GRK4	-0.73	3.0E-03
RNF19B	0.58	3.0E-03
FN3K	-1.24	3.0E-03
SOCS3	1.25	3.0E-03
ANPEP	1.58	3.0E-03
PRR22	-1.14	3.0E-03
RASL11B	0.73	3.1E-03
WDR45	-0.61	3.1E-03
TMEM41B	0.58	3.1E-03
CCDC53	-0.62	3.1E-03

TTC3P1	-1.09	3.1E-03
QPCTL	0.62	3.2E-03
KCTD21	-0.89	3.2E-03
CHST2	0.90	3.2E-03
HAPLN3	0.58	3.2E-03
NR0B1	1.18	3.3E-03
APOLD1	-0.67	3.3E-03
ROMO1	0.60	3.3E-03
LINC00648	-1.21	3.3E-03
C1orf145	-1.17	3.4E-03
BSCL2	-0.90	3.4E-03
RGMA	0.60	3.4E-03
CABLES2	-0.59	3.4E-03
ZNF385C	-1.01	3.4E-03
MCF2L	-0.61	3.4E-03
PDXP	1.63	3.4E-03
ZNF337-AS1	-0.87	3.4E-03
PDK4	0.76	3.4E-03
CCDC28B	0.65	3.4E-03
RUSC1-AS1	-1.08	3.4E-03
ALPK1	-0.59	3.5E-03
FAM86EP	-0.99	3.5E-03
PARK2	-0.82	3.5E-03
MC1R	-1.06	3.6E-03
ALG5	-0.67	3.6E-03
CDH10	-0.72	3.6E-03
DENND2C	0.59	3.6E-03
PRKAR1B	-0.63	3.6E-03
CLHC1	-0.76	3.6E-03
FHOD1	0.60	3.7E-03
DNAJB9	0.68	3.7E-03
NXT2	-0.61	3.7E-03
SLC35B3	-0.58	3.7E-03
LRRC57	0.62	3.8E-03
ZNF34	0.74	3.8E-03
KIAA1456	-1.08	3.8E-03
MARVELD3	0.70	3.9E-03
PKD2	-0.64	3.9E-03
CACNA2D3	0.80	3.9E-03
SLC6A16	-0.73	3.9E-03
ZSCAN30	-0.58	3.9E-03
EPN1	0.58	3.9E-03
TCIRG1	-1.18	4.0E-03
KIAA1024	-0.58	4.0E-03
SPATA18	-0.64	4.0E-03
NPPC	1.04	4.0E-03
CCDC176	-1.10	4.0E-03

IRX3	0.68	4.0E-03
SOS1-IT1	0.81	4.0E-03
LINC00467	-0.73	4.1E-03
ZNF485	-0.77	4.1E-03
FRY	0.59	4.2E-03
SNHG17	0.60	4.2E-03
EXTL3-AS1	0.93	4.3E-03
NPTX1	-0.61	4.3E-03
H1FX	0.59	4.3E-03
FAM83H-AS1	-0.80	4.3E-03
NPL	-0.64	4.3E-03
CRY2	0.62	4.4E-03
RNASET2	0.64	4.4E-03
GKAP1	0.63	4.5E-03
B3GAT2	-0.78	4.5E-03
C8orf48	-1.45	4.5E-03
TFEB	0.82	4.5E-03
GNB2	0.70	4.6E-03
MTG1	-0.70	4.6E-03
METAP1D	-0.63	4.6E-03
ZNF284	0.70	4.6E-03
MBD3	-0.59	4.6E-03
CLEC11A	1.07	4.6E-03
FOXD3	0.97	4.8E-03
ADRB1	1.10	4.8E-03
RNF170	-0.61	4.8E-03
FAM226A	1.15	4.9E-03
FAM226B	1.15	4.9E-03
RHBDL1	-1.00	4.9E-03
SSR4P1	-1.64	4.9E-03
RAB27B	-0.82	5.0E-03
NELFB	0.59	5.1E-03
PTMS	0.61	5.1E-03
HSCB	0.77	5.1E-03
PRR4	-1.03	5.1E-03
C6orf183	-0.59	5.2E-03
ZNF10	-0.65	5.2E-03
KRTCAP2	0.66	5.2E-03
KCTD18	-0.59	5.2E-03
WDR96	-1.46	5.2E-03
KCNJ4	0.96	5.2E-03
FILIP1L	-1.46	5.2E-03
ZNF382	-0.70	5.2E-03
PLAG1	0.68	5.3E-03
PILRA	-1.09	5.4E-03
RHPN1	-0.98	5.4E-03
OMA1	-0.61	5.4E-03

FOXN4	-0.74	5.4E-03
PITX1	0.61	5.4E-03
ABCC8	-0.72	5.5E-03
ZBED3-AS1	-0.90	5.5E-03
SNORD94	1.01	5.5E-03
PTGES2	0.74	5.6E-03
SLC23A3	-1.63	5.7E-03
EPM2A	-0.65	5.7E-03
PHLDA1	0.66	5.7E-03
IFT88	-0.59	5.8E-03
GOLGA2P5	-0.74	5.8E-03
SNORD101	1.46	5.9E-03
CCDC180	-1.71	5.9E-03
C19orf52	0.61	6.0E-03
SLC25A34	-1.55	6.0E-03
PAXIP1-AS1	0.76	6.0E-03
ABCG1	0.99	6.1E-03
LSMEM1	1.25	6.1E-03
SNORD83A	0.90	6.1E-03
TGM2	-0.85	6.1E-03
HOXA10-AS	-0.66	6.2E-03
ZSCAN12P1	-1.27	6.2E-03
WEE2-AS1	-1.25	6.2E-03
C1orf50	0.62	6.2E-03
FAM50B	-0.71	6.2E-03
CDO1	-0.64	6.3E-03
GLIPR1	-0.73	6.4E-03
ZNF611	-0.65	6.4E-03
OSGEPL1	-0.59	6.4E-03
MAP2K6	-0.61	6.4E-03
MID1IP1-AS1	-1.15	6.4E-03
DMTN	-0.93	6.5E-03
SEMA3G	-0.79	6.6E-03
TEX35	-1.41	6.6E-03
RERG	-1.18	6.7E-03
PRR13	0.59	6.7E-03
NKX2-5	0.81	6.7E-03
PCBD2	-0.76	6.8E-03
HSD17B7	0.76	6.8E-03
WDR81	-0.67	6.8E-03
EMC9	0.64	6.9E-03
EFCAB6	-1.10	6.9E-03
C6orf165	0.92	6.9E-03
C6orf163	-1.21	6.9E-03
SPINK5	-1.04	6.9E-03
LINC00526	1.27	7.0E-03
SCARNA10	-1.32	7.2E-03

GTPBP10	-0.66	7.2E-03
REPS2	-0.69	7.4E-03
ST3GAL2	0.58	7.4E-03
IQCG	-0.69	7.4E-03
FAM195A	0.84	7.4E-03
EIF3J-AS1	0.70	7.4E-03
ARL6IP6	-0.61	7.5E-03
MGLL	0.81	7.5E-03
SCUBE1	-0.59	7.5E-03
LINC01123	1.11	7.5E-03
ORAI2	0.58	7.5E-03
C11orf70	-0.79	7.5E-03
SRSF8	-0.71	7.6E-03
STEAP1	-0.62	7.7E-03
TPI1P2	-1.60	7.7E-03
TTC32	-0.59	7.7E-03
HIST1H1C	0.74	7.7E-03
FAM86C2P	-0.65	7.8E-03
PNPLA3	0.64	7.8E-03
BEST1	-1.00	7.8E-03
TRIM59	0.82	7.8E-03
ZDHHC1	-0.74	7.9E-03
C19orf10	0.68	7.9E-03
IFT80	-0.60	7.9E-03
QDPR	0.64	7.9E-03
TSPAN2	-0.69	7.9E-03
BHLHB9	-0.73	8.0E-03
PALM2	0.73	8.2E-03
BCL7C	0.61	8.3E-03
SNORD69	0.85	8.3E-03
PTGER2	-1.17	8.6E-03
JAKMIP1	-1.09	8.7E-03
CLDN4	0.92	8.7E-03
CARD9	-1.39	8.8E-03
BCL10	0.58	8.8E-03
EMILIN2	0.60	8.8E-03
FAM149A	-0.68	8.9E-03
MAP6	-1.04	8.9E-03
NME5	-0.93	9.0E-03
REG1A	1.01	9.0E-03
MBOAT1	0.63	9.1E-03
BIN3	-0.58	9.1E-03
HSD3B7	-1.25	9.2E-03
NRG4	-0.60	9.2E-03
PRR7	1.09	9.3E-03
SNORD20	1.45	9.3E-03
IFI27	-0.82	9.3E-03

ELMO3	-1.11	9.3E-03
C16orf46	1.34	9.4E-03
FAM96B	0.58	9.4E-03
CYP2D6	-1.24	9.4E-03
BRAF	0.67	9.5E-03
RGS9	-0.87	9.6E-03
CACTIN-AS1	-1.17	9.6E-03
ZBED5-AS1	-1.16	9.6E-03
GABRA5	-0.59	9.6E-03
FAM174B	-0.78	9.6E-03
NDUFC2	-0.78	9.7E-03
SYNPO2	-1.49	9.8E-03
LOH12CR1	0.58	9.9E-03
ICA1L	-0.65	9.9E-03
SMIM4	0.87	9.9E-03
FOLR1	-0.91	9.9E-03
DLK2	1.22	9.9E-03
TMEM99	-0.59	1.0E-02
TCHH	-0.83	1.0E-02
CCT6P3	0.67	1.0E-02
CCDC147-AS1	1.11	1.0E-02
MED31	-0.59	1.0E-02
ZBTB11-AS1	-1.02	1.0E-02
CNR1	-1.24	1.0E-02
RELL2	0.62	1.0E-02
C10orf10	0.72	1.0E-02
KCTD17	0.58	1.1E-02
DNAJC5G	-1.36	1.1E-02
HILPDA	-0.69	1.1E-02
C1orf116	1.01	1.1E-02
RPL18AP3	1.44	1.1E-02
MT2A	0.70	1.1E-02
FERMT1	-1.36	1.1E-02
STK31	-1.19	1.1E-02
EHBP1L1	-0.60	1.1E-02
UBE2V1	0.83	1.1E-02
INE1	0.81	1.1E-02
TMEM17	-0.89	1.1E-02
SYTL4	0.63	1.1E-02
KIAA0391	0.97	1.1E-02
C7orf63	0.72	1.1E-02
SNORA33	1.30	1.1E-02
KCNC1	-0.65	1.1E-02
BMP8B	-0.63	1.1E-02
FAM132B	-0.58	1.1E-02
ZBTB7C	-0.63	1.1E-02
BMP4	0.59	1.2E-02

EXOSC4	0.76	1.2E-02
ZNF32-AS2	0.98	1.2E-02
NAT14	0.60	1.2E-02
CHL1	-0.95	1.2E-02
CXXC4	-0.60	1.2E-02
TMEM51-AS1	-1.20	1.2E-02
IER5L	1.19	1.2E-02
SPSB1	-0.68	1.2E-02
STYK1	0.75	1.2E-02
PPAN	-0.89	1.2E-02
CDH7	-0.58	1.2E-02
INCA1	-1.31	1.3E-02
MRPS31P5	0.98	1.3E-02
LRRTM4	-0.74	1.3E-02
PLEKHF1	0.76	1.3E-02
DMPK	-0.62	1.3E-02
SPAG17	-1.43	1.3E-02
CCDC7	-0.99	1.3E-02
HRSP12	-0.61	1.3E-02
CCDC178	-1.32	1.3E-02
ANKRD24	-0.79	1.3E-02
WAS	-1.21	1.3E-02
PLEKHN1	1.03	1.3E-02
ZNF707	0.61	1.3E-02
TMEM27	-1.14	1.3E-02
PDE7B	-1.22	1.3E-02
HCG15	-0.85	1.3E-02
SMN2	-0.67	1.4E-02
CDK15	-0.84	1.4E-02
SNORD17	0.72	1.4E-02
PLIN1	-0.88	1.4E-02
LY6G5B	-0.75	1.4E-02
PABPC1P3	1.12	1.4E-02
DUOX2	-1.33	1.4E-02
SNRNP25	0.65	1.4E-02
FGF5	-0.80	1.4E-02
FLVCR1-AS1	0.90	1.4E-02
ANKLE1	-1.07	1.4E-02
IL12RB2	-0.63	1.4E-02
ZBTB8B	0.66	1.4E-02
CYP27B1	-0.65	1.5E-02
NDST4	-1.23	1.5E-02
TMEM54	0.92	1.5E-02
CAMK2B	-1.17	1.5E-02
PTPRB	-0.82	1.5E-02
LURAP1	0.74	1.5E-02
POF1B	-1.26	1.5E-02

DRP2	0.73	1.5E-02
PPP1R3F	-0.82	1.5E-02
PINK1-AS	-0.67	1.5E-02
SNX18P3	-0.83	1.5E-02
HSPB1	0.59	1.5E-02
CCDC183-AS1	-0.69	1.5E-02
HBQ1	1.25	1.5E-02
SPSB3	0.60	1.5E-02
APLF	-0.83	1.5E-02
MIR4519	0.97	1.5E-02
BTBD19	-0.71	1.5E-02
HYPK	0.63	1.5E-02
FAM131B	0.77	1.6E-02
NACAD	-0.91	1.6E-02
PSMD6-AS2	-0.99	1.6E-02
TMEM169	-1.37	1.6E-02
HNRNPA3P6	1.06	1.6E-02
ARMC12	-1.28	1.6E-02
KLF15	0.66	1.6E-02
LINC00909	-0.77	1.6E-02
ANKRD20A4	0.93	1.7E-02
SAMD15	-1.02	1.7E-02
CAPN10-AS1	0.85	1.7E-02
ID1	0.88	1.7E-02
GOLGA8R	-0.79	1.7E-02
LINC01021	-0.75	1.7E-02
PPAPDC1A	-0.87	1.7E-02
AMIGO1	-0.73	1.7E-02
XK	-0.69	1.7E-02
CYBRD1	-0.64	1.7E-02
HES4	0.68	1.7E-02
WDR72	-0.71	1.7E-02
C15orf59	-0.66	1.7E-02
COL24A1	-0.67	1.7E-02
EPPK1	-0.81	1.7E-02
CHRNB1	0.79	1.7E-02
RPS15A	0.63	1.8E-02
CTNNA3	-0.84	1.8E-02
ZNF628	0.80	1.8E-02
ZNF28	-0.61	1.8E-02
CYP3A5	-0.75	1.8E-02
OTUD1	0.64	1.8E-02
CCDC122	-0.61	1.8E-02
RRAS	0.78	1.9E-02
CLYBL	-0.62	1.9E-02
CYP2J2	-0.69	1.9E-02
LINC01003	1.27	1.9E-02

NEUROG2	1.16	1.9E-02
TMEM155	-1.05	1.9E-02
GLIS1	0.69	1.9E-02
MTUS2	1.21	1.9E-02
TECTA	-0.85	1.9E-02
SLC8A3	-0.82	1.9E-02
CEP44	0.67	1.9E-02
IFITM3	0.84	2.0E-02
TMEM44-AS1	-0.98	2.0E-02
RNF180	-0.86	2.0E-02
T	-1.26	2.0E-02
CYP4F26P	-0.73	2.0E-02
TGFB1	0.72	2.0E-02
CCDC181	0.69	2.0E-02
BCL3	0.78	2.0E-02
C9orf16	0.63	2.0E-02
SLC26A4	-1.24	2.0E-02
NOTCH2NL	-0.59	2.1E-02
USP2-AS1	1.02	2.1E-02
TBC1D10A	0.68	2.1E-02
TNS1	-0.63	2.1E-02
DDX47	0.77	2.1E-02
NRAS	1.16	2.1E-02
ABCA8	-0.68	2.1E-02
SLC46A3	-0.70	2.1E-02
OCEL1	0.68	2.1E-02
MKX	-0.62	2.1E-02
GJB7	-0.63	2.1E-02
SLC52A2	0.59	2.2E-02
MAPK13	0.77	2.2E-02
ARHGEF37	-0.66	2.2E-02
ZNF805	0.62	2.2E-02
RDH14	-0.65	2.2E-02
PDE3A	-0.80	2.2E-02
BFSP1	-0.98	2.2E-02
IFIT2	-1.13	2.2E-02
RAB4B	0.84	2.2E-02
BHLHE41	-0.62	2.3E-02
KLRAP1	-0.67	2.3E-02
PLA2G7	-0.61	2.3E-02
C2CD4C	-1.09	2.3E-02
LINC01116	-1.03	2.3E-02
KCNE4	-0.98	2.3E-02
BBS5	0.66	2.3E-02
CEL	-1.02	2.4E-02
KRT8P12	0.65	2.4E-02
SLC17A7	1.09	2.4E-02

GIPR	0.76	2.4E-02
ZNF17	-0.58	2.4E-02
GPR137C	-0.58	2.4E-02
RGS5	-0.61	2.4E-02
LIN7B	0.59	2.4E-02
TTC25	-0.93	2.4E-02
B3GALT5	-0.68	2.4E-02
HSPB6	-1.19	2.4E-02
UNC5A	-0.91	2.4E-02
STEAP1B	-0.65	2.4E-02
CCDC107	0.66	2.4E-02
RIBC2	-0.67	2.4E-02
MAFB	0.65	2.5E-02
MSRA	0.92	2.5E-02
SPRY2	0.59	2.5E-02
TMEM126A	0.66	2.5E-02
NDNF	-0.66	2.5E-02
CRIP3	0.91	2.5E-02
TSACC	-0.75	2.6E-02
LRRIQ3	-1.21	2.6E-02
CCDC144CP	-0.70	2.6E-02
RFTN2	-1.13	2.6E-02
U2AF1L4	0.68	2.6E-02
FAM66D	-1.25	2.7E-02
SBF2-AS1	-0.76	2.7E-02
SYNGR4	-0.99	2.7E-02
SUV420H2	0.64	2.7E-02
KNDC1	-0.77	2.7E-02
CXorf24	1.06	2.8E-02
CUBN	-0.85	2.8E-02
MIR1254-1	0.83	2.8E-02
ZNF582	-0.64	2.8E-02
CELSR3	-0.59	2.8E-02
FAM157A	0.62	2.8E-02
DIO2	-0.97	2.9E-02
LRRN2	-1.09	2.9E-02
LINC00893	-0.75	2.9E-02
ADRA2C	0.59	2.9E-02
ZCCHC18	0.95	2.9E-02
RDH16	0.89	2.9E-02
LRRTM2	-0.78	2.9E-02
GLDN	-0.61	2.9E-02
NEB	-1.06	3.0E-02
LAMTOR5-AS1	-0.84	3.0E-02
GLUD1P3	0.70	3.0E-02
TMCC1-AS1	-0.92	3.0E-02
TRPM3	-0.93	3.0E-02

PIK3IP1	0.71	3.0E-02
KCNJ11	1.01	3.0E-02
HLX	0.61	3.1E-02
CCDC144A	-1.03	3.1E-02
OVOL2	0.95	3.1E-02
EOMES	-0.61	3.1E-02
WDR78	-0.92	3.1E-02
MUC20	-1.11	3.2E-02
ARIH2OS	1.13	3.2E-02
CDHR3	-0.67	3.2E-02
GATA6-AS1	0.73	3.2E-02
PTH1R	-1.19	3.2E-02
SH2D4A	-1.19	3.2E-02
SCARNA17	-1.28	3.2E-02
PCSK1N	0.90	3.2E-02
DDX60	-0.59	3.2E-02
RPL28	0.59	3.2E-02
FOXP3	-1.18	3.2E-02
FAM203A	-0.66	3.2E-02
DNAJC22	-0.58	3.3E-02
ZNF846	-0.77	3.3E-02
DNAJC30	-0.62	3.4E-02
FER1L4	-0.75	3.4E-02
MRPS17	-0.72	3.5E-02
ATP5D	0.60	3.5E-02
LRRC69	-1.07	3.5E-02
URAHP	0.81	3.6E-02
SMCO4	0.69	3.6E-02
MIF	0.59	3.6E-02
DNAAF3	-0.78	3.6E-02
LINC00461	-0.84	3.6E-02
INA	-0.68	3.6E-02
TMEM184A	-1.20	3.6E-02
ZC3H6	0.58	3.7E-02
zfhx2-as1	-0.87	3.7E-02
CYP4F32P	-0.73	3.7E-02
SNORA66	1.02	3.8E-02
FRMD3	-1.03	3.8E-02
TMEM145	-0.59	3.8E-02
SPIN3	-0.63	3.9E-02
MLKL	-0.88	4.0E-02
ZNF429	-0.60	4.0E-02
SENP8	-0.59	4.1E-02
EFCAB4B	-0.66	4.1E-02
SRRM5	-0.81	4.1E-02
ZNF571-AS1	-0.80	4.1E-02
COL9A1	-0.61	4.2E-02

C1orf213	0.72	4.2E-02
SRRM2-AS1	-0.62	4.3E-02
RMDN2	-1.00	4.3E-02
AKAP3	-0.92	4.3E-02
SLC2A4	0.69	4.3E-02
SNX18P16	-1.08	4.4E-02
GPR3	0.85	4.4E-02
CCNT2-AS1	-0.86	4.4E-02
PCDH1	0.61	4.4E-02
ADPRHL1	-0.62	4.5E-02
CPLX1	0.66	4.5E-02
RXRG	-0.94	4.5E-02
ZMYND10	-0.86	4.5E-02
NAIP	-0.82	4.5E-02
EMR2	-0.66	4.6E-02
GPC2	0.61	4.7E-02
CDK5R2	1.07	4.7E-02
C12orf60	-0.79	4.7E-02
SNORA50	0.92	4.7E-02
NIPSNAP3B	0.73	4.7E-02
COL4A4	-0.69	4.8E-02
ZNF23	0.75	4.9E-02
ACRC	-0.64	5.0E-02
CAPS2	-1.22	5.0E-02
