# Supplementary material for: Epigenetic Vulnerability of Insulator CTCF Motifs at Parkinson’s Disease-Associated Genes in Response to Neurotoxicant Rotenone
Source: Front Genet. 2020 Jul 7;11:627. doi: 10.3389/fgene.2020.00627 (PMC7381335; doi:10.3389/fgene.2020.00627)
Supplement: Supplementary file 3 [file Data_Sheet_3.PDF]

Supplemental Tables and Figures:

Supplemental Table 1. qPCR primers for RNAseq Validation

PARK2	GTGTTTGTCAGGTTCAACTCCA
	GAAAATCACACGCAACTGGTC
ITGA8	AGAATGGAGACCTTATTGTGGGA
	GAGCCACTTCCGTCTGCTTT
CHCHD2	ACACATTGGGTCACGCCATTA
	GCACCTCATTGAAACCCTCACA
GPRIN3	AGGGCACCTCCACCATTTG
	GACACTAACACGCCGAAGTCA
FER	TGCTTCTGGACTCCTTACAAAAG
	TTAGCAGCCGTTGTTCTGTGA
CNSKR3	GACTGCCTGCAACAATATGTCC
	CTGGTGTCCAATCCGTGTGAC
UBOX5	CAAGTGGTGTTTAGCCACAGG
	GAAAGAGCCCCTTTATTCCAGAG
BMP4	ATGATTCCTGGTAACCGAATGC
	CCCCGTCTCAGGTATCAAACT
WNT3	AGGGCACCTCCACCATTTG
	GACACTAACACGCCGAAGTCA

Supplemental Table 2. Bisulfite PCR primers

CHCHD2	TTTTATATTTTTTTAATATAGGTTTTA<br>AATAAACCACAAAAACTTCC
CNKSR3	GTTTATATGTGGTTTGAAAATGTAG<br>AAAAATAAAAATAACCTTTCTATAAC
UBOX5	TTGAAGTTTTTATTATAGTTAGGTTTG<br>TCCAAAAAAATATTTTCCCTCTACA
ITGA8	GTATTTGGAATATTTAGGATTTG<br>CTAAATAACAACCACCCACC
PARK2-CTCF	TTTAGTTATAGTTTTGTTGGAAGGA<br>CAAAAACACAAAAAACAAAAAAACA
GPRIN3	TAAAGGATTTTTGTGTAAAATGTGG<br>AAACTCTCACCTCCAAACTAACTCTAC
FER	TTAGTTTAGGGTTTAAGTTTTGTTTT<br>CCCAACCAAAAATACTCTACTAC
BMP4	TTATTTTTTTTGGATTTTAGAGT<br>CTAAATATCTAACTTATCTCCCC

Supplemental Table 3. ChIP-qPCR primers

BMP4	CAGGTAGCCTTGCTCACCAT<br>CCGGAAGCTAGGTGAGTGTG
UBOX5	CTGAAGTTCTCACCACAGCCA<br>GAGGAGCCAGTATCTGTGTCG
GPRIN3	TCGCATATCCCAAGCACCG<br>ATGAACAGTCGGGCAAGTGA
FER	TCAGGTTCTAGGTAGGTGCGT<br>GGGAGGATGAGCGGATGAC
CNKSR3	CCTGGCGCAAATGCTATGG<br>CCGAGCCTGTCTGTTTTTGTT
PARK2	CCTGCTGCTTTGAGCCTTTTT<br>TCAAAGGCTGTTGCTTGCTT
CHCHD2	ACGTTCAATCTACCCCCGC<br>TCTACTGGGGCAATGACGC

Supplemental Table 4. Top Pathway Enrichment from Reactome Pathways

Pathway name	Entities				Reactions	
	found	ratio	p-value	FDR*	found	ratio
SMAD2/SMAD3:SMAD4 heterotrimer regulates transcription	5 / 39	0.003	7.12e-04	0.235	3 / 17	0.001
Pre-NOTCH Transcription and Translation	7 / 89	0.006	0.001	0.235	13 / 28	0.002
Interaction With Cumulus Cells And The Zona Pellucida	3 / 12	8.31e-04	0.001	0.235	2 / 2	1.60e-04
TP53 Regulates Transcription of Genes Involved in Cytochrome C Release	4 / 33	0.002	0.003	0.316	4 / 25	0.002
Transcriptional activity of SMAD2/SMAD3:SMAD4 heterotrimer	5 / 55	0.004	0.003	0.316	4 / 38	0.003
Pre-NOTCH Expression and Processing	7 / 113	0.008	0.004	0.366	13 / 38	0.003

Supplemental Table 5. Molecular Function Gene Ontology

GO term	Description	P-value	FDR q-value
GO:0003676	nucleic acid binding	3.06E-08	5.35E-05
GO:0097159	organic cyclic compound binding	6.06E-07	5.30E-04
GO:1901363	heterocyclic compound binding	7.10E-07	4.14E-04
GO:0003723	RNA binding	1.11E-05	4.87E-03
GO:0003674	molecular_function	1.16E-04	4.07E-02
GO:0003677	DNA binding	4.88E-04	1.42E-01
GO:0044389	ubiquitin-like protein ligase binding	6.36E-04	1.59E-01

Supplemental Table 6. Cellular Component Gene Ontology

GO term	Description	P-value	FDR q-value
GO:0005654	nucleoplasm	2.40E-07	2.38E-04
GO:0044428	nuclear part	4.09E-06	2.03E-03
GO:0005634	nucleus	8.33E-06	2.76E-03
GO:0043231	intracellular membrane-bounded organelle	1.05E-05	2.61E-03
GO:0044424	intracellular part	1.86E-05	3.70E-03
GO:0043227	membrane-bounded organelle	1.05E-04	1.74E-02
GO:0043229	intracellular organelle	1.27E-04	1.80E-02
GO:0005786	signal recognition particle, endoplasmic reticulum targeting	2.89E-04	3.59E-02
GO:0048500	signal recognition particle	2.89E-04	3.19E-02

GO:0043226	organelle	3.52E-04	3.50E-02
GO:0044446	intracellular organelle part	9.92E-04	8.95E-02

Supplemental Table 7. Differentially Methylated Cytosine at all CpG sites

GENE_CHR	start	end	strand	pvalue	qvalue	meth.diff
chr14_54422869_54423420_BMP4	483	483	+	5.48E-08	5.60E-08	-3.27
chr20_3140226_3140678_UBOX5	194	194	+	2.59E-27	9.41E-27	2.28
chr20_3140226_3140678_UBOX5	203	203	+	3.46E-34	1.62E-33	3.57
chr20_3140226_3140678_UBOX5	42	42	+	4.51E-55	4.42E-54	1.61
chr20_3140226_3140678_UBOX5	47	47	+	3.67E-35	1.89E-34	-1.11
chr20_3140226_3140678_UBOX5	63	63	+	1.29E-41	9.75E-41	1.75
chr20_3140226_3140678_UBOX5	67	67	+	2.54E-63	3.12E-62	2.14
chr20_3140226_3140678_UBOX5	111	111	+	8.44E-70	1.18E-68	-1.44
chr4_90228647_90229070_GPRIN3	45	45	+	2.37E-07	2.24E-07	-1.08
chr4_90228647_90229070_GPRIN3	53	53	+	1.87E-13	3.06E-13	1.32
chr4_90228647_90229070_GPRIN3	55	55	+	3.42E-13	5.41E-13	1.19
chr4_90228647_90229070_GPRIN3	62	62	+	1.23E-33	5.49E-33	-2.55
chr4_90228647_90229070_GPRIN3	106	106	+	2.38E-09	2.82E-09	1.13
chr4_90228647_90229070_GPRIN3	114	114	+	1.33E-13	2.29E-13	1.68
chr4_90228647_90229070_GPRIN3	117	117	+	4.10E-07	3.73E-07	1.02
chr4_90228647_90229070_GPRIN3	145	145	+	2.58E-11	3.61E-11	-1.21
chr4_90228647_90229070_GPRIN3	175	175	+	4.03E-16	8.78E-16	1.65
chr4_90228647_90229070_GPRIN3	202	202	+	1.47E-36	8.03E-36	2.71
chr4_90228647_90229070_GPRIN3	213	213	+	3.23E-44	2.88E-43	1.85
chr4_90228647_90229070_GPRIN3	235	235	+	2.11E-16	4.71E-16	-1.22
chr4_90228647_90229070_GPRIN3	241	241	+	8.83E-14	1.55E-13	1.26
chr4_90228647_90229070_GPRIN3	253	253	+	4.35E-10	5.54E-10	1.03
chr4_90228647_90229070_GPRIN3	294	294	+	6.21E-22	1.90E-21	1.07
chr4_90228647_90229070_GPRIN3	297	297	+	5.85E-14	1.06E-13	-1.09
chr4_90228647_90229070_GPRIN3	319	319	+	1.52E-27	5.98E-27	2.29
chr4_90228647_90229070_GPRIN3	339	339	+	2.04E-11	2.91E-11	1.29
chr4_90228647_90229070_GPRIN3	351	351	+	4.97E-21	1.43E-20	1.84
chr4_90228647_90229070_GPRIN3	354	354	+	5.16E-13	7.91E-13	1.89
chr5_108084418_108084954_FER	133	133	+	1.53E-06	1.26E-06	-1.59
chr5_108084418_108084954_FER	143	143	+	5.23E-76	8.56E-75	-4.38
chr5_108084418_108084954_FER	163	163	+	1.62E-140	1.59E-138	-4.94
chr5_108084418_108084954_FER	197	197	+	2.40E-14	4.62E-14	-1.33
chr5_108084418_108084954_FER	222	222	+	2.79E-12	4.14E-12	1.62
chr5_108084418_108084954_FER	271	271	+	1.05E-16	2.39E-16	1.88
chr5_108084418_108084954_FER	274	274	+	1.12E-19	2.98E-19	3.37
chr5_108084418_108084954_FER	277	277	+	1.23E-11	1.79E-11	-2.80
chr5_108084418_108084954_FER	280	280	+	1.56E-15	3.26E-15	1.69
chr5_108084418_108084954_FER	297	297	+	4.73E-03	2.52E-03	-1.82
chr5_108084418_108084954_FER	366	366	+	5.38E-07	4.77E-07	1.89
chr5_108084418_108084954_FER	376	376	+	3.76E-04	2.32E-04	1.33
chr5_108084418_108084954_FER	380	380	+	2.60E-04	1.67E-04	-1.70

chr5_108084418_108084954_FER	387	387	+	1.36E-15	2.90E-15	2.73
chr5_108084418_108084954_FER	454	454	+	2.37E-07	2.24E-07	2.11
chr5_108084418_108084954_FER	476	476	+	2.26E-03	1.28E-03	-1.39
chr5_108084418_108084954_FER	22	22	+	2.39E-05	1.73E-05	1.41
chr5_108084418_108084954_FER	70	70	+	2.15E-10	2.78E-10	3.89
chr5_108084418_108084954_FER	74	74	+	5.92E-21	1.66E-20	5.16
chr5_108084418_108084954_FER	84	84	+	1.16E-08	1.26E-08	-2.73
chr5_108084418_108084954_FER	116	116	+	1.23E-04	8.50E-05	-1.59
chr5_108084418_108084954_FER	126	126	+	1.64E-27	6.17E-27	-4.02
chr6_154830537_154830958_CNKSR3	6	6	+	8.22E-22	2.44E-21	-2.48
chr6_154830537_154830958_CNKSR3	47	47	+	1.66E-24	5.63E-24	1.81
chr6_154830537_154830958_CNKSR3	233	233	+	2.43E-59	2.65E-58	-1.46
chr6_154830537_154830958_CNKSR3	273	273	+	1.02E-43	8.35E-43	1.66
chr6_154830537_154830958_CNKSR3	280	280	+	3.60E-14	6.66E-14	-1.08
chr6_154830537_154830958_CNKSR3	300	300	+	1.41E-93	6.93E-92	3.29
chr6_154830537_154830958_CNKSR3	326	326	+	8.54E-20	2.33E-19	-1.04
chr6_163277806_163278291_CTCF	35	35	+	3.89E-04	2.38E-04	-1.68
chr6_163277806_163278291_CTCF	137	137	+	8.27E-07	7.12E-07	-1.96
chr7_56173886_56174373_CHCHD2	130	130	+	1.67E-08	1.76E-08	-1.11
chr7_56173886_56174373_CHCHD2	147	147	+	4.52E-04	2.75E-04	-1.91
chr7_56173886_56174373_CHCHD2	217	217	+	1.71E-05	1.29E-05	1.66
chr7_56173886_56174373_CHCHD2	221	221	+	1.01E-08	1.11E-08	-1.08
chr7_56173886_56174373_CHCHD2	263	263	+	2.07E-04	1.37E-04	1.56
chr7_56173886_56174373_CHCHD2	293	293	+	3.24E-04	2.05E-04	1.20
chr7_56173886_56174373_CHCHD2	60	60	+	7.91E-07	6.87E-07	1.73
chr7_56173886_56174373_CHCHD2	64	64	+	6.04E-05	4.29E-05	-1.27
chr7_56173886_56174373_CHCHD2	100	100	+	1.55E-11	2.23E-11	1.86

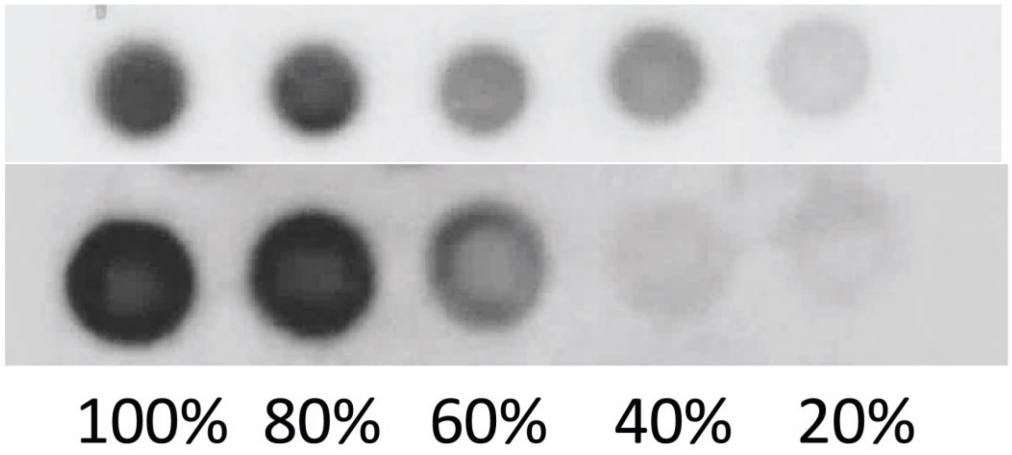

Supplemental Figure 1. DNA methylation standard control ladder with 100% 5mC.

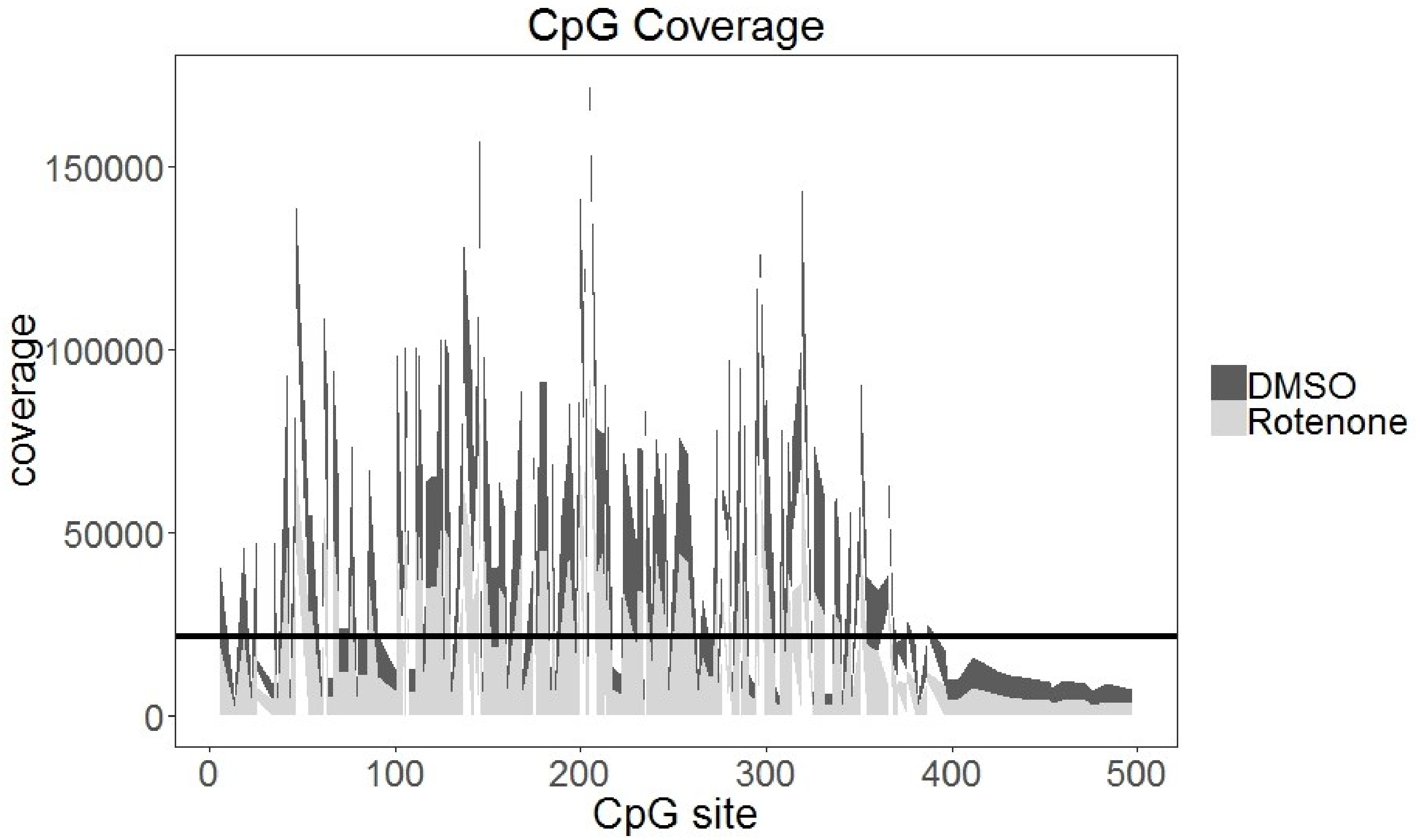

Supplementary Figure 2. Bisulfite sequencing coverage of CpG sites within amplified regions at Parkinson's disease genes. The average total coverage for all CpG sites within the amplified region is indicated by the straight line.
